# Microbiome-metabolome signatures in mice genetically prone to develop dementia, fed a normal or fatty diet

**DOI:** 10.1038/s41598-018-23261-1

**Published:** 2018-03-20

**Authors:** Elena Sanguinetti, Maria Carmen Collado, Vannina G. Marrachelli, Daniel Monleon, Marta Selma-Royo, Mercedes M. Pardo-Tendero, Silvia Burchielli, Patricia Iozzo

**Affiliations:** 1 Institute of Clinical Physiology, National Research Council, 56124 Pisa, Italy; 2 Institute of Agrochemistry and Food Technology, National Research Council, 46980 Paterna Valencia, Spain; 3 Health Research Institute, INCLIVA, 46010 Valencia, Spain; 4 Gabriele Monasterio Tuscany Foundation, 56124 Pisa, Italy; 5 Department of Pathology, University of, Valencia, Spain

## Abstract

Cognitive decline, obesity and gut dysfunction or microbial dysbiosis occur in association. Our aim was to identify gut microbiota-metabolomics signatures preceding dementia in genetically prone (3xtg) mice, with and without superimposed high-fat diet. We examined the composition and diversity of their gut microbiota, and serum and faecal metabolites. 3xtg mice showed brain hypometabolism typical of pre-demented stage, and lacked the physiological bacterial diversity between caecum and colon seen in controls. Cluster analyses revealed distinct profiles of microbiota, and serum and fecal metabolome across groups. Elevation in Firmicutes-to-Bacteroidetes abundance, and exclusive presence of *Turicibacteraceae*, *Christensenellaceae*, *Anaeroplasmataceae* and *Ruminococcaceae*, and lack of *Bifidobacteriaceae*, were also observed. Metabolome analysis revealed a deficiency in unsaturated fatty acids and choline, and an overabundance in ketone bodies, lactate, amino acids, TMA and TMAO in 3xtg mice, with additive effects of high-fat diet. These metabolic alterations were correlated with high prevalence of *Enterococcaceae*, *Staphylococcus*, *Roseburia*, *Coprobacillus* and *Dorea*, and low prevalence of *S24.7*, *rc4.4* and *Bifidobacterium*, which in turn related to cognitive impairment and cerebral hypometabolism. Our results indicate an effect of transgenic background on gut microbiome-metabolome, enhanced by high-fat diet. The resulting profiles may precede overt cognitive impairment, suggesting their predictive or risk-stratifying potential.

## Introduction

Abnormalities in the intestinal microbiota have been described in association with numerous diseases, most frequently obesity and bowel inflammation^1,2^. Recent evidence suggests that the microbiota may be strongly involved in the pathogenesis of neuropathologies^3^. Among them, psychiatric conditions, autism and Parkinson’s disease have received most attention, whereas there seem to be no studies addressing Alzheimer’s disease (AD) and dementia. These epidemic diseases are growing in prevalence at an alarming rate, fostered by population aging, and by an increasing frequency and severity of predisposing conditions^4^. Recent findings document that in mice and humans with AD, neurodegenerative changes occur in the gut, similar to the brain^5,6^. Obesity is among commonest risk factors for cognitive decline and AD, and high-fat diet has been shown to accelerate cognitive deterioration, especially in genetically predisposed models^7,8^. High-fat diets are known to modify gut function and bacteria composition^9^.

Microbiota-mediated signals can reach the brain via neural and circulating routes^10^. The microbiota regulates the transport of nutrients across the gut barrier, and produces absorbable or non-absorbable metabolites, affecting circulating and faecal metabolic profiles^11,12^. These can modulate systemic inflammation, i.e. a hallmark of obesity and neurodegenerative diseases, or cross the blood-brain barrier to act directly on the brain^12–14^. Metabolites, whose synthesis, modification or absorption have been ascribed to the microbiota include lipids and lipoproteins, amino acids, glutamate, choline, acetate, butyrate, glycerol and modulators of inflammation and oxidative stress, which are considered crucial in regulating neurological development and preservation^15–23^.

In spite of the notion that the gastrointestinal environment varies profoundly from the proximal to the distal end of the intestine, affecting bacteria composition, most of the available knowledge is based on faecal bacteria obtained from the very end of the intestine^24^.

Here, we postulated that the combined characterization of microbiome, and circulating and faecal metabolome might reveal signatures and provide mechanistic insight related to neurodegenerative disease, and its accentuation by high-fat feeding. We studied 3xtg transgenic mice at an early cerebral disease stage and control mice, both undergoing normal or high-fat feeding. We examined their caecum and colon microbiota composition and diversity (within and between intestinal segments), and also metabolites in faeces (caecum and colon) and serum. *In vivo* cerebral metabolism and cognitive function were assessed to confirm the subclinical disease stage.

## Results

### General characterization

We characterized n = 45 mice, grouped in normal diet-fed (ND, n = 9), high-fat diet-fed (HFD, n = 9), ND-fed triple transgenic (3xtg, n = 8) and HFD-fed 3xtg mice (3xtg-HFD, n = 7). Diet was administered up to 8 months of age, when body weight, cognitive performance by Y-maze test, and brain glucose metabolism by ^18^F-2-fluoro-2-deoxyglucose positron emission tomography/computed tomography ([^18^F]FDG PET/CT) were assessed. At the end of the dieting period, 8-month-old HFD mice were heavier than ND mice (51.8 ± 2.6 vs 41.7 ± 1.5, p < 0.01; growth curves, Fig. S1), whereas a tendency was shown in 3xtg-HFD vs 3xtg mice (39.7 ± 1.8 vs 35.5 ± 1.0, p = 0.1). As per design, we aimed to target the pre-demented stage, thus the Y-maze test showed a non-significant ∼20–40% cognitive decline in 3xtg models. Two [^18^F]FDG PET/CT images of control ND mice were excluded due to persistent hyperglycaemia on the imaging day. A marked (∼30–40%) reduction in cerebral [^18^F]FDG extraction was observed in 3xtg and 3xtg-HFD mice compared to controls, especially in the hippocampus and temporal cortex, consistent with a pre-demented state (Table 1).

**Table 1:** Regional cerebral glucose metabolism as determined by [^18^F]FDG-PET.

Standardized Uptake Value (%ID/g*mmol/l)	ND	HFD	3xtg	3xtg-HFD
Hippocampus	2.93 ± 0.25	2.70 ± 0.36	1.87 ± 0.14^**,^^	1.80 ± 0.19^**,^^
Frontal cortex	2.51 ± 0.23	2.61 ± 0.37	1.73 ± 0.16^^^	1.77 ± 0.23^^^
Temporal cortex	2.36 ± 0.12	2.47 ± 0.31	1.56 ± 0.16^*,^^^	1.41 ± 0.14^**,^^^

Mann-Withney U statistical test. Data are mean ± standard error of the mean.

*p < 0.05. **p < 0.01: 3xTg and 3xTg-HFD vs ND.

^p < 0.05. ^^p < 0.01: 3xTg and 3xTg-HFD vs HFD.

### Impact on the metabolome

**Figure 1:**
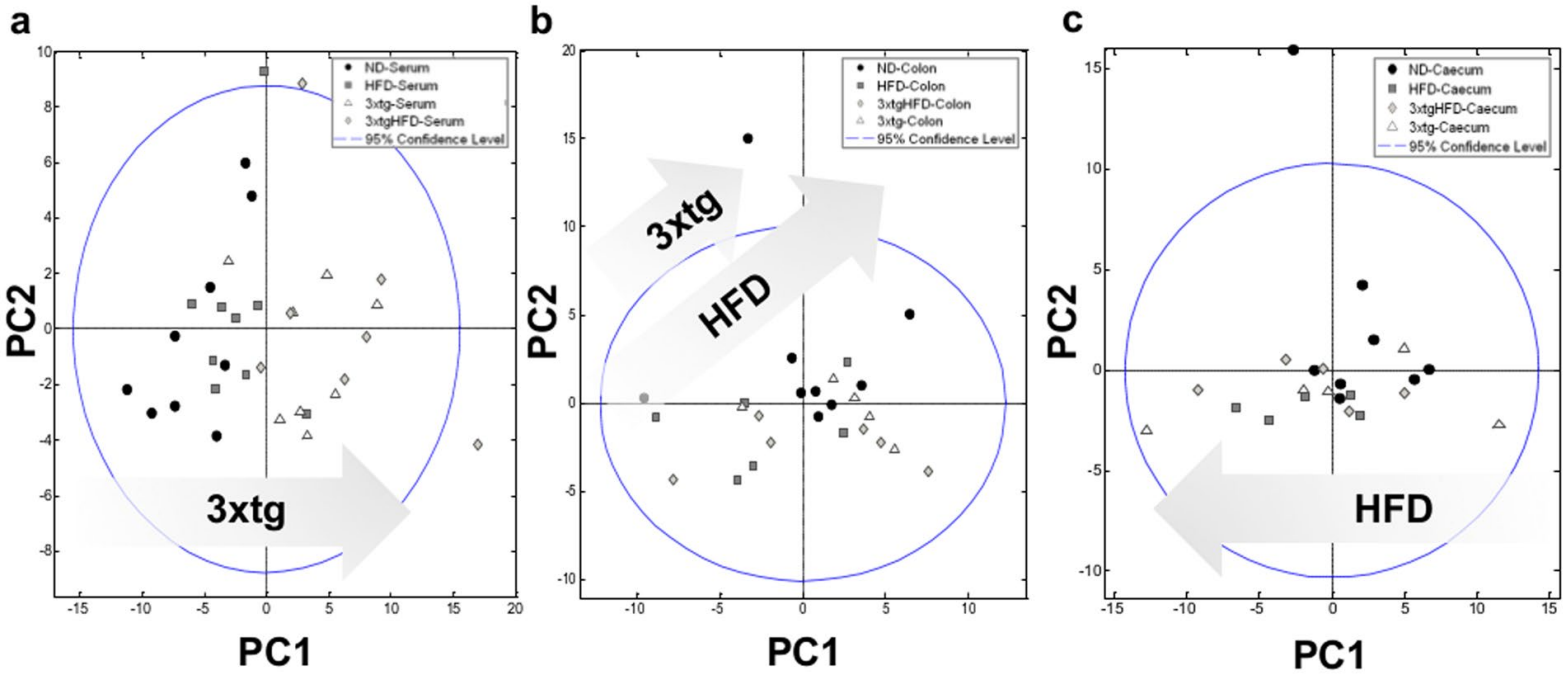
The metabolome of 3xtg mice differs more from control mice than mice under HFD. PCA score plots based on the NMR metabolomic profile of serum (**a**), and fecal extracts from colon (**b**) and caecum (**c**). Each symbol represents a single sample shaped and coloured according to the respective group.

**Figure 2:**
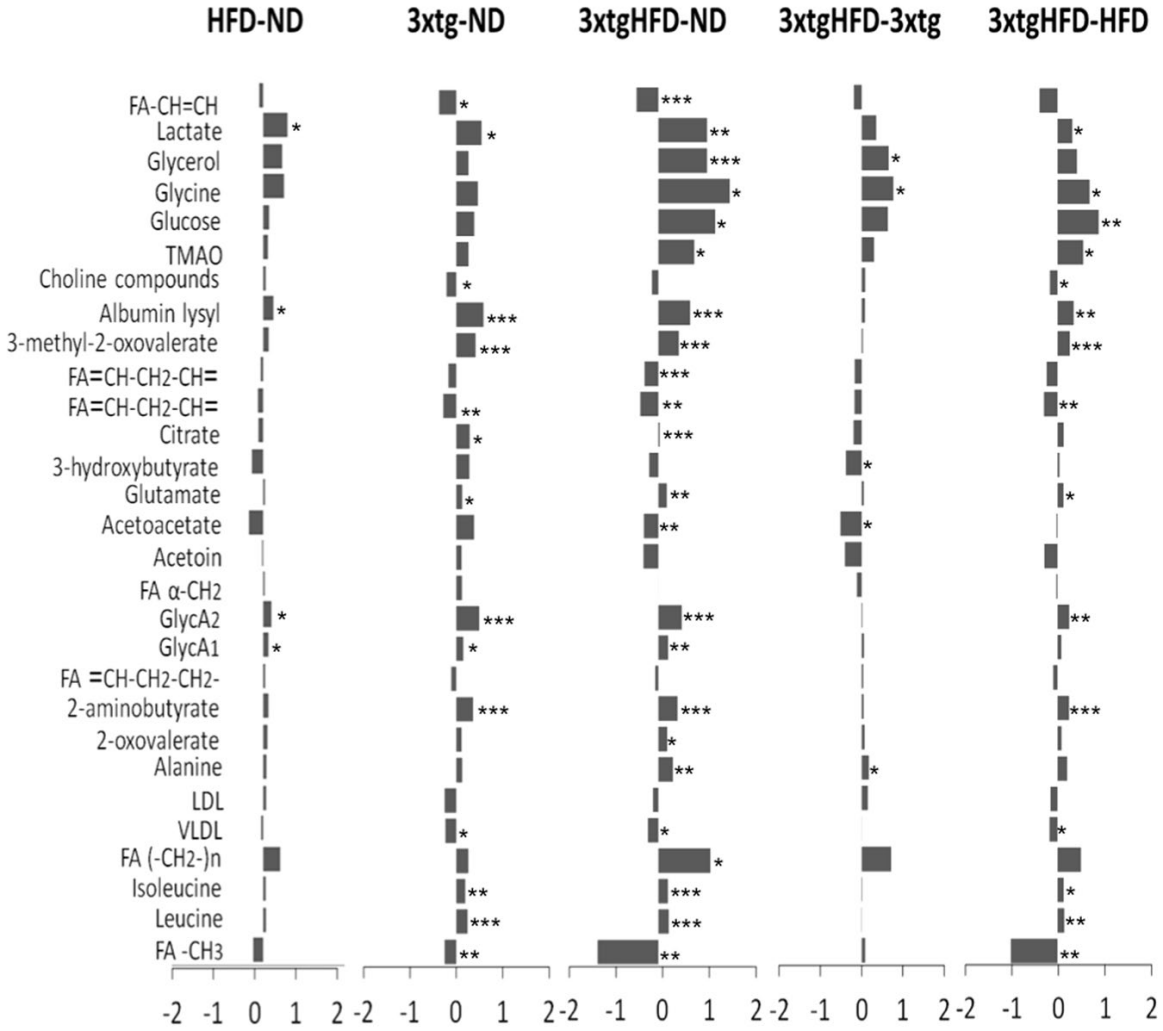
The serum metabolome of 3xtg mice differs from control mice, and HFD exerts additive effects. Relative fold changes for control and 3xtg mice under normal or HFD on NMR integrals for metabolic regions with higher VIP scores in PLS-DA serum analysis. Bars represent fold-change in metabolite content for each comparison (increased content: positive bars; decreased content: negative bars). ND, ND-fed control mice; 3xtg, ND-fed 3xtg mice; HFD, HFD-fed control mice; 3xtgHFD, HFD-fed 3xtg mice. *p < 0.05; **p < 0.01; ***p < 0.001.

**Figure 3:**
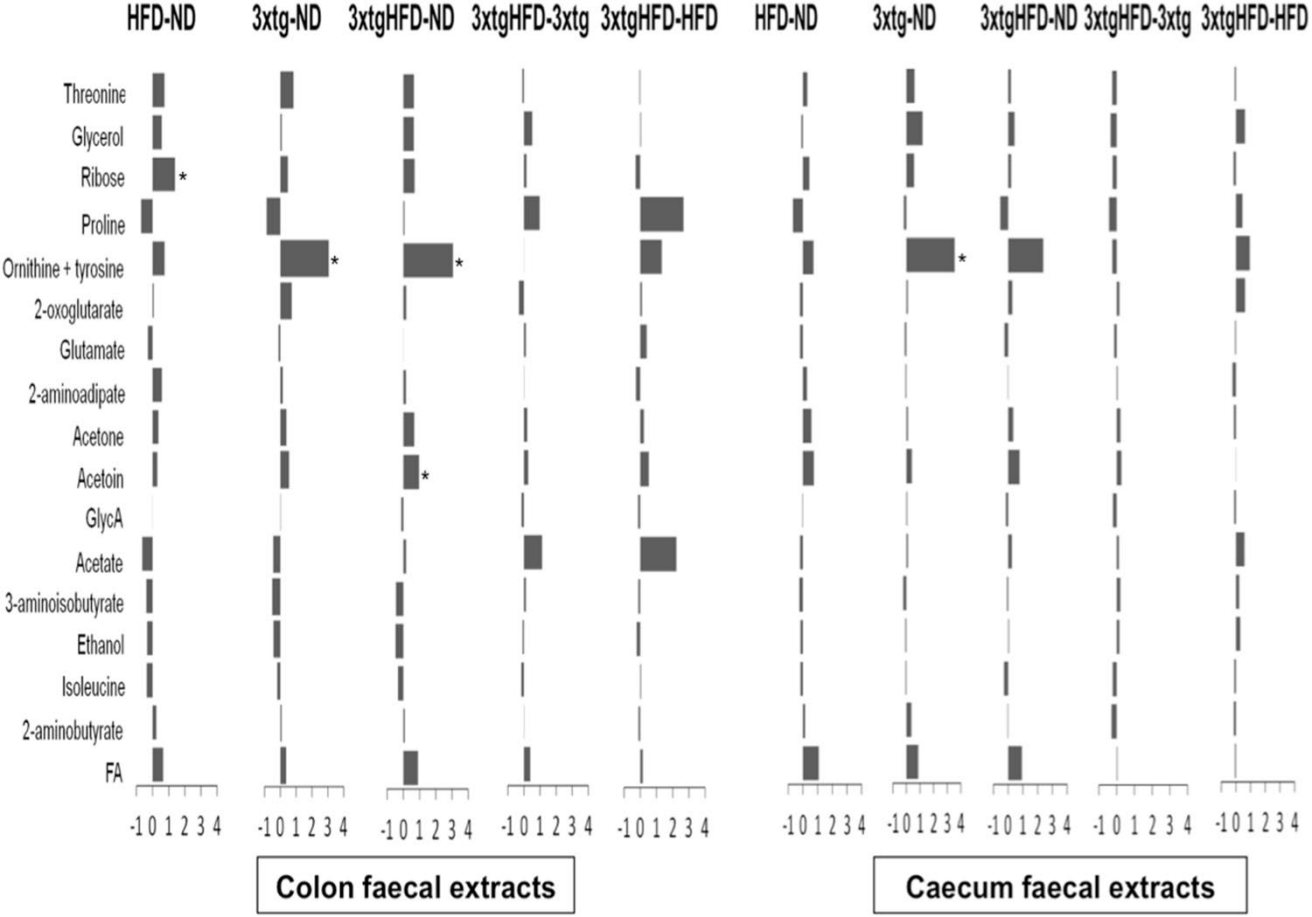
The fecal metabolome of 3xtg mice is altered with respect to control mice. Relative fold changes for in test groups (HFD, and 3xtg mice under ND or HFD) versus control ND mice, derived from NMR integrals of metabolic regions with higher VIP scores in the PLS-DA analysis in colon and caecum faecal extracts. Bars represent fold-change in metabolite content in each comparison (increased content: positive bars; decreased content: negative bars). ND, ND-fed control mice; 3xtg, ND-fed 3xtg mice; HFD, HFD-fed control mice; 3xtgHFD, HFD-fed 3xtg mice. *p < 0.05; **p < 0.01; ***p < 0.001.

We quantified 77 and 99 metabolites in all serum and faecal (caecum and colon) samples, respectively (Tables S1-2), and built chemometric models for detecting global and individual metabolic trends (Fig. 1, Figs S2–5). Serum analysis by Principal Component Analysis (PCA) showed distinct clusters in 3xtg compared to control mice, indicating genotype-induced changes in metabolome (Fig. 1a). More specifically, we observed a clear separation along the first Principal Component (PC1, 46.54% of variance) between 3xtg and control mice, regardless of diet, suggesting a stronger metabolic impact of 3xtg genotype than HFD. Serum metabolites explaining this separation pattern are given in the PCA loading plot (Fig. S2). Metabolites and samples segregation in response to diet and disease were attenuated at faecal level, where a greater effect of HFD compared to 3xtg genotype was observed in the determination of samples segregation (Fig. 1b,c). To further explore the metabolomic relationships between 3xtg genotype and diet, we built separated partial least-squares discriminant analysis (PLS-DA) models discriminating 3xtg and control mice, under ND or HFD (Figs S3–5). These plots highlighted a strong separation between 3xtg and controls in both serum and fecal extracts, regardless of diet, and revealed greater HFD-mediated separation in 3xtg compared to control mice. For serum and faecal metabolites, we quantified relative fold-changes in all possible binary comparisons to evaluate the extent of metabolomic variations in the different groups (Figs 2–3), thus observing that both 3xtg genotype and HFD affect some metabolites in the same direction, but to a different extent, prevailing for the 3xtg background.

#### Impact of diet

A significant increase in inflammatory marker (glycoprotein acetylation biomarker, GlycA), lactate and albumin lysil serum levels was observed in HFD compared to ND mice (Fig. 2). At faecal level, group comparisons did not show regional differences between colon and caecum tracts (Fig. 3) and a large interindividual variation in metabolic profiles seemed to attenuate group effects. A tendency towards higher ribose, threonine, threonine-ornithyne, glycerol and fatty acid levels, and lower proline, acetate and ethanol amounts was observed in HFD compared to control mice (especially in colon), but only colon ribose levels achieved statistical significance.

#### Impact of disease

The 3xtg genotype induced strong metabolic changes in host (serum) metabolome, but minor changes in host-microbial co-metabolome (faeces) (Figs 2–3). Differences in serum metabolome of 3xtg compared to control mice, regardless of diet, included elevation in inflammatory markers (GlycA), lactate, albumin lysil, leucine and isoleucine, and deficient levels of unsaturated fatty acids, very low density lipoprotein (VLDL), and choline. In addition, higher levels of trimethylamine (TMA) and a tendency to trimethylamine N-oxide (TMAO) elevation occurred in 3xtg mice compared to controls. At faecal level, 3xtg mice were characterized by a pronounced increase in ornithine-tyrosine levels compared to controls, in both colon and caecum.

#### Combined impact of diet and disease

The combined effect of HFD and 3xtg genotype amplified the changes observed in each component alone. As expected, the comparison of 3xtg genotype under HFD vs control animals under ND showed the largest number of statically significant differences and fold changes (Fig. 2). Similar to 3xtg mice, high aminoacids (leucine, isoleucine, glycine) and albumin lysil serum levels were detected compared to controls. The amount of systemic inflammatory markers (GlycA) was elevated, but not higher than in 3xtg under ND, due to already high inflammatory levels in this group. Instead, a further elevation in glucose, lactate, glycerol and glutamate levels was observed compared to controls and to 3xtg under ND. Likewise, fatty acid (but not VLDL) deficiencies were also amplified in 3xtg-HFD, compared to each condition (3xtg or HFD) alone. Finally, TMA and TMAO levels were progressively greater from ND to 3xtg and to 3xtg-HFD. Important, the combination of HFD and 3xtg genotype affected ketone bodies pathways in an alternative way compared to the above listed metabolites, due to the opposing effects of HFD, lowering and 3xtg, elevating acetoacetate and 3-hydroxibutyrate. The net effect resulted in a significant reduction of ketone bodies in 3xtg-HFD mice compared to controls. In faeces, ornithine-threonine and acetoin levels were significantly higher in the colon of 3xtg-HFD compared to ND mice (Fig. 3).

### Impact on the microbiome

A total of 2,895,848 quality-filtered 16 S rDNA sequences were obtained from caecum and colon faecal extracts (52,651.8 ± 34,735.2 seq/sample) and clustered into 23,874 Operational Taxonomic Units (OTUs).

#### Impact of location

Analysis of Similarity (ANOSIM) test showed significant differences at OTU level between colon and caecum microbiome (R = 0.184, p = 0.039). In group analysis, they were significant in ND (R = 0.184, p = 0.043) but not in 3xtg mice (R = 0.007, p = 0.412).

Higher Bacteroidetes (27.57% vs 13.0%, p = 0.0016, p Bonferroni correction = 0.0096, false discovery rate, FDR = 0.0075) and lower Firmicutes (64.41% vs 81.97%, p = 0.0025, p Bonferroni correction = 0.015, FDR = 0.0075) were present in colon compared to caecum samples in ND mice. However, no differences at phylum level were observed between colon and caecum samples in the 3xtg group.

At family level, caecum samples showed higher levels of *Lachnospiraceae* (18.02% vs 10.28%, p = 0.036) and lower levels of *S24.7* (13% vs 27.56%, p = 0.0016) than colon samples in ND. Conversely, only *Enterococcaceae* family was significantly different between colon and caecum samples in 3xtg mice (2.31% vs 0.09%, respectively, p = 0.012). In linear discriminant analysis effect size (LEfSe) tests, *S24.7* family was significantly enriched in ND colon compared to caecum samples (linear discriminant analysis, LDA, score > 5, p < 0.05), while *Enterococcaceae* family was enriched in 3xtg colon compared to caecum samples (LDA score = 4.5, p < 0.05). At OTU level, we found significant differences in 30 OTU belonging mostly to *Firmicutes* and *Bacteroidetes* phyla when ND colon and caecum samples were compared, while only 8 OTU significantly differed in 3xtg. Higher abundance of *Oscillospira* (p = 0.035) and lower abundance of *Enterococcus* spp. (p = 0.05) genera was detected in caecum compared to colon samples in 3xtg (data not shown).

Higher Shannon diversity index (p = 0.022), but no differences in microbial richness measured by Chao1 index (p = 0.512), was observed in caecum compared to colon samples in ND. Conversely, no alpha diversity (Shannon and Chao1 indexes), was reported between colon and caecum samples in 3xtg mice (Fig. S6).

#### Impact of diet

**Figure 4:**
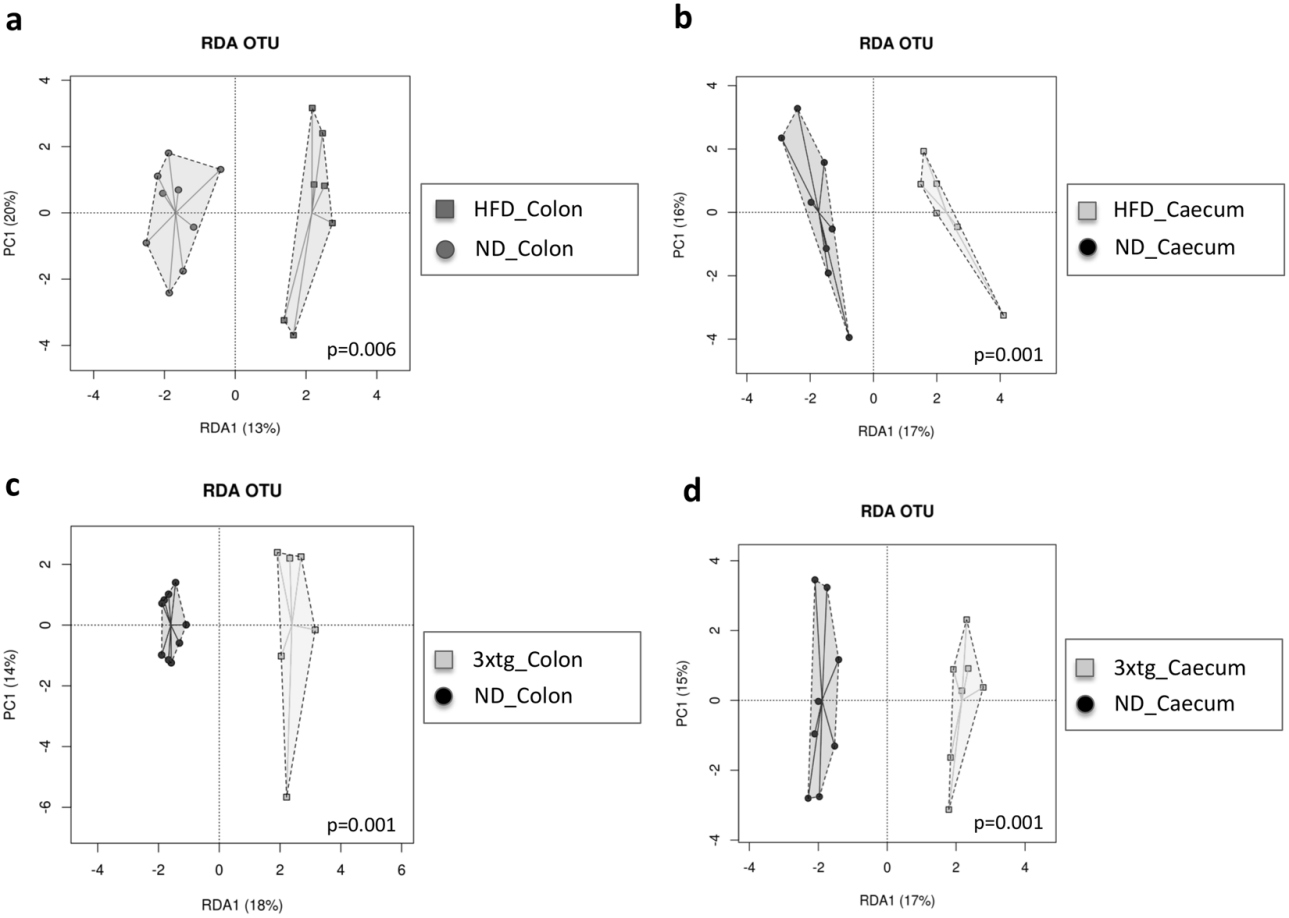
HFD and 3xtg determine a dramatic rightward shift in the overall composition of gut microbiota. RDA plot at OTU level between animal groups and diet intervention. The analysis revealed a clear-cut separation between HFD and ND mice (**a**,**b**), and between 3xtg and control mice (**c**,**d**). Each symbol represents a single sample shaped and coloured according to the respective group.

Redundancy Analysis (RDA) showed significant differences in microbial composition between HFD and ND mice, in colon (p = 0.006) and caecum samples (p = 0.001) (Fig. 4b), as confirmed by ANOSIM tests (colon: R = 0.209, p = 0.023; caecum: R = 0.213, p = 0.032).

At family level, lower abundance of *Bifidobacteriaceae* (p = 0.029), *Lactobacillaceae* (p = 0.071) and *S24.7* (p = 0.055) and higher abundance of *Rikenellaceae* (p = 0.0013, FDR = 0.023), *Mogibacteriaceae*. (p = 0.0074, FDR = 0.074), *Lachnospiraceae* (p = 0.012, FDR = 0.072) *Enterococcaceae* (p = 0.022, FDR = 0.09), were detected in HFD compared to ND colon samples. Similar results were found in caecum samples, where lower levels of *Bifidobacteriaceae* (p = 0.063) and *S24.7* (*p* = 0.029), and higher abundance of *Rikenellaceae* (p = 0.00077, FDR = 0.013), *Enterococcaceae* (p = 0.0088, FDR = 0.052) and S*taphylococcaceae* (p = 0.0092, FDR = 0.052) were observed in the HFD compared to ND group.

At genus level, lower abundance of *Unclassified Peptococcaceae* (p = 0.013), *Bifidobacterium* and *Lactobacillus* spp (p = 0.029 and 0.072, respectively) and higher abundance of *Unclassified Rikenellaceae* (p = 0.0013, FDR = 0.042), *Unclassified Mogibacteriaceae* (p = 0.007), and *Unclassified Enterococcaceae* (p = 0.022) were observed in HFD compared to ND colon samples.

DESeq 2 test confirmed diet-based differences between groups, specifically lower abundance of *Bifidobacterium* and *Lactobacillus* were found in HFD than ND mice (p < 0.0001, FDR < 0.0001 for both). In caecum samples, we also observed lower abundance of *Bifidobacterium* spp (p = 0.036) and *Ruminococcus* (p = 0.043), and higher abundance of *Staphylococcus* (p = 0.009) and *Clostridium* spp (p = 0.07) genera in HFD compared to ND mice. Most significant findings in pooled samples (caecum and colon) are shown in Supplementary Figure 7.

**Figure 5:**
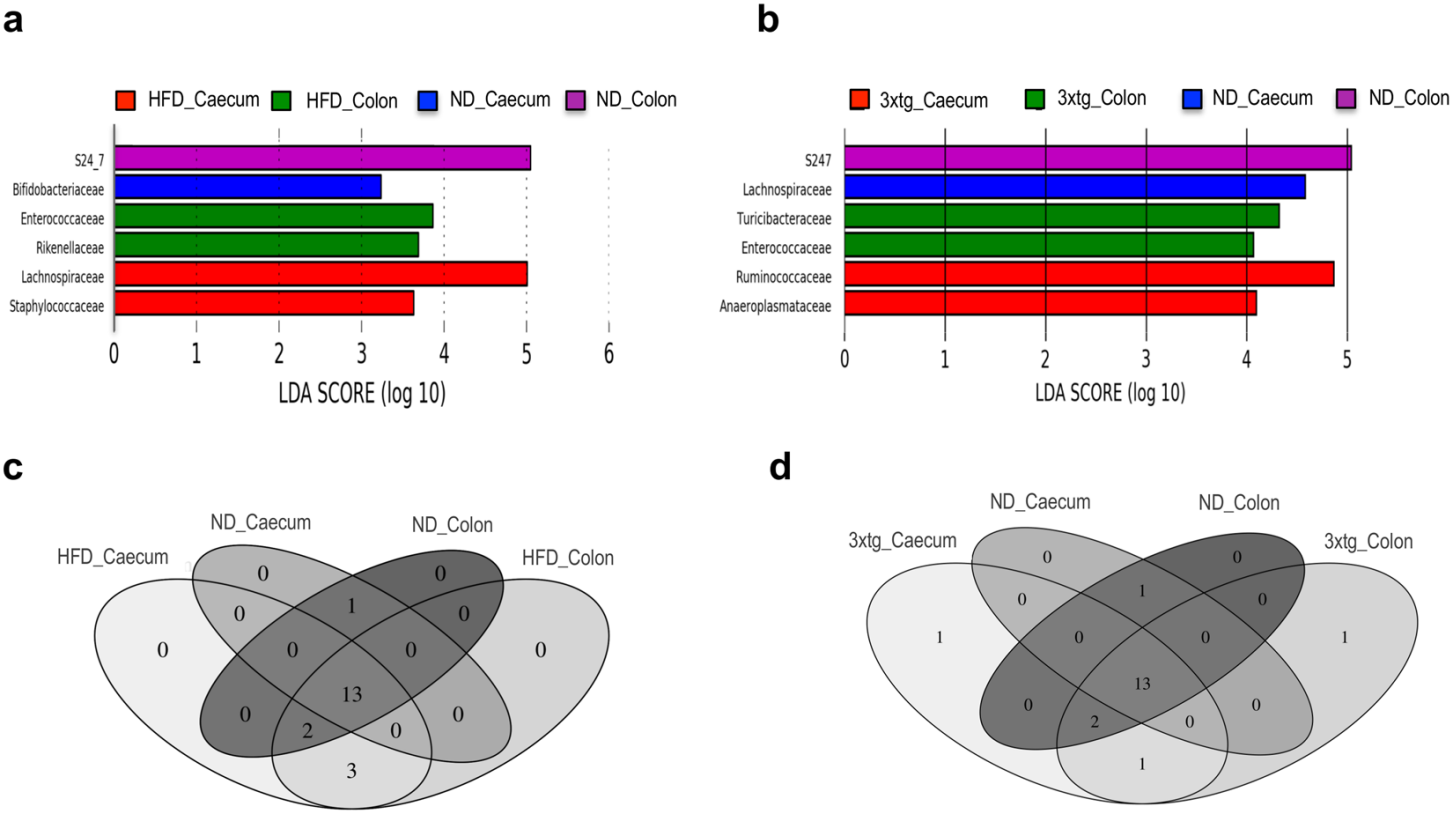
HFD consumption, 3xtg background and location significantly influence the composition of the gut microbiome. LEfSe test-identified LDA scores showed the significant bacterial difference due to the effect of diet and location (**a**, ND and HFD, colon and caecum), and disease and location (**b**, ND and 3xtg, colon and caecum). Venn diagram showed shared families across groups (**c**, diet and location; **d**, disease and location).

At OTU level, we found significant differences in 37 OTU belonging mostly to Firmicutes, Bacteroidetes and Actinobacteria phyla (Table S3) in colon, and also 37 OTU in caecum when ND and HFD groups were compared (Table S4). No differences in Shannon diversity index and lower Chao1 richness index (p = 0.086, colon; p = 0.0223, caecum) were observed in HFD compared to ND groups (Fig. S6). LEfSe tests revealed that at family level in ND mice, *S24.7* was significantly enriched in colon, while *Bifidobacteriaceae* were highly abundant in caecum (Fig. 5a). In HFD mice, *Enterococcaceae* and *Rikenellaceae* were representative of colon, whereas *Lachnospiraceae* and *Staphylococcaecae* were abundant in caecum samples. Venn diagram showed a core of 13 families shared between diet groups and locations (Fig. 5c). *Bifidobacteriaceae* family was exclusively present in ND colon and caecum. *Christensenellaceae*, *Mogibacteriaceae* and *Rikenellaceae* were present in HFD colon and caecum. *Staphylococcaceae* and *Enterococcaceae*, were shared between ND colon and HFD colon and caecum.

#### Impact of disease

RDA showed differences in the microbial composition of colon (p = 0.001) and caecum samples (p = 0.001), between ND and 3xtg (Fig. 4c,d), as confirmed by ANOSIM tests (colon: R = 0.696, p = 0.001; caecum: R = 0.326, p = 0.001).

At family level, lower abundance of *S24.7* (p = 0.018) and higher abundance of *Enterococcaceae* (p = 0.0077, FDR = 0.081) and *Turicibacteraceae* (p = 0.0081, FDR = 0.081) were detected in 3xtg compared to ND colon samples. Similar results were found in caecum samples, in which lower levels of *S24.7* (p = 0.029) and *Bifidobacteriaceae* (p = 0.045) were detected in 3xtg compared to ND mice. At genus level, lower abundance of *Bifidobacterium* spp (p = 0.045) and *rc4.4* (p = 0.042), and higher abundance of *Roseburia* (p = 0.049) were detected in 3xtg compared to ND caecum. LEfSe analyses confirmed that, at family level *S24.7* and *Lachnospiraceae* was significantly enriched in ND colon and caecum, respectively, while *Enterococcaceae* and *Turicibacteriaceae* were associated to 3xtg colon samples (Fig. 5b). Families belonging to *Ruminococcaceae* and *Anaeroplasmataceae* were representative of 3xtg caecum samples. At genus level, *Turicibacter* and *Staphylococcus* were significantly enriched in 3xtg colon samples, whereas *Anaeroplasma* was associated to 3xtg caecum samples. Most significant findings in pooled samples (caecum and colon) are shown in Supplementary Figure 7.

Venn diagram showed a core of 13 families shared between 3xtg and control mice in both locations (Fig. 5d). *Turicibacteraceae* family was associated to 3xtg colon, while *Christensenellaceae* to 3xtg caecum. *Bifidobacteriaceae* was related to ND colon and caecum, whereas *Anaeroplasmataceae* was linked to 3xtg colon and caecum. Finally, *Staphylococcaceae* and *Enterococcaceae* families were shared between ND colon and 3xtg colon and caecum.

At OTU level, we found significant differences in 48 OTU (Table S5) in colon and 62 OTU in caecum (Table S6) when ND and 3xtg groups were compared. Alpha diversity analysis showed no group differences in Shannon diversity index, but lower Chao1 richness index (p = 0.0195) in 3xtg than ND colon samples (Fig. S6). No significant difference was found in caecum, though the tendency was the same.

#### Impact of combined location, diet and disease

**Figure 6:**
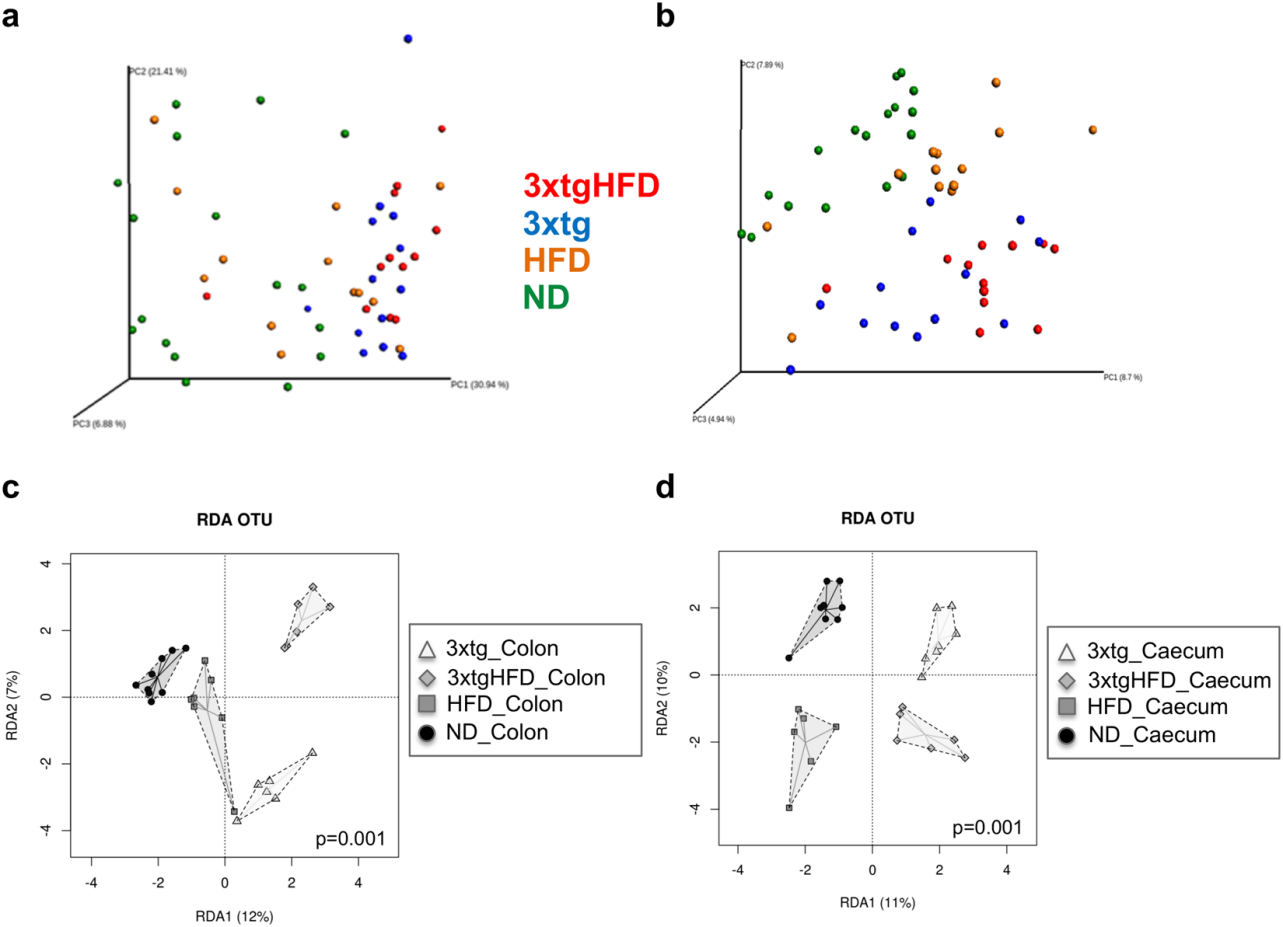
Isolated and combined HFD plus 3xtg background determine a clear-cut separation in microbiota composition. Beta-diversity PCA using weighted (**a**) and unweighted-UniFrac distances (**b**). RDA plot at OTU level between groups (**c**, colon; **d**, caecum). Each symbol represents a single sample shaped and coloured according to the respective group.

Principal Coordinates Analysis (PCoA) of the samples clearly separated 3xtg from control animals, in both weighted (Fig. 6a) and unweighed (Fig. 6b) plots (PC1 accounting for 8.7% and 30.94% of the variance in unweighted and weighted UniFrac analysis, respectively), indicating that the microbiota between groups is compositionally distinct. In addition, the different diet groups are well defined and clustered. ANOSIM tests confirmed the overall significant difference between groups and intestinal locations (R = 0.477, p = 0.001). Similar results were found when microbiome of ND, HDF, 3xtg and 3xtg-HFD groups in each intestinal location (colon: R = 0.53, p = 0.001; caecum: R = 0.589, p = 0.001) were compared.

Together with UniFrac data, ADONIS (permutational manova, PERMANOVA) showed significant differences at OTU level between diet groups and sample location (p = 0.0006). In addition, RDA highlighted significant differences in the microbial composition between the diets and disease status in colon (p = 0.001) and caecum (p = 0.001) (Fig. 6c,d).

Alpha diversity analysis showed no differences in Shannon diversity index, but higher Chao1 richness index (p < 0.0001 in colon and p = 0.0256 in caecum) between groups and locations, as higher richness was found in ND followed by HFD group (Fig. S6).

### Microbiome and metabolome associations

**Figure 7:**
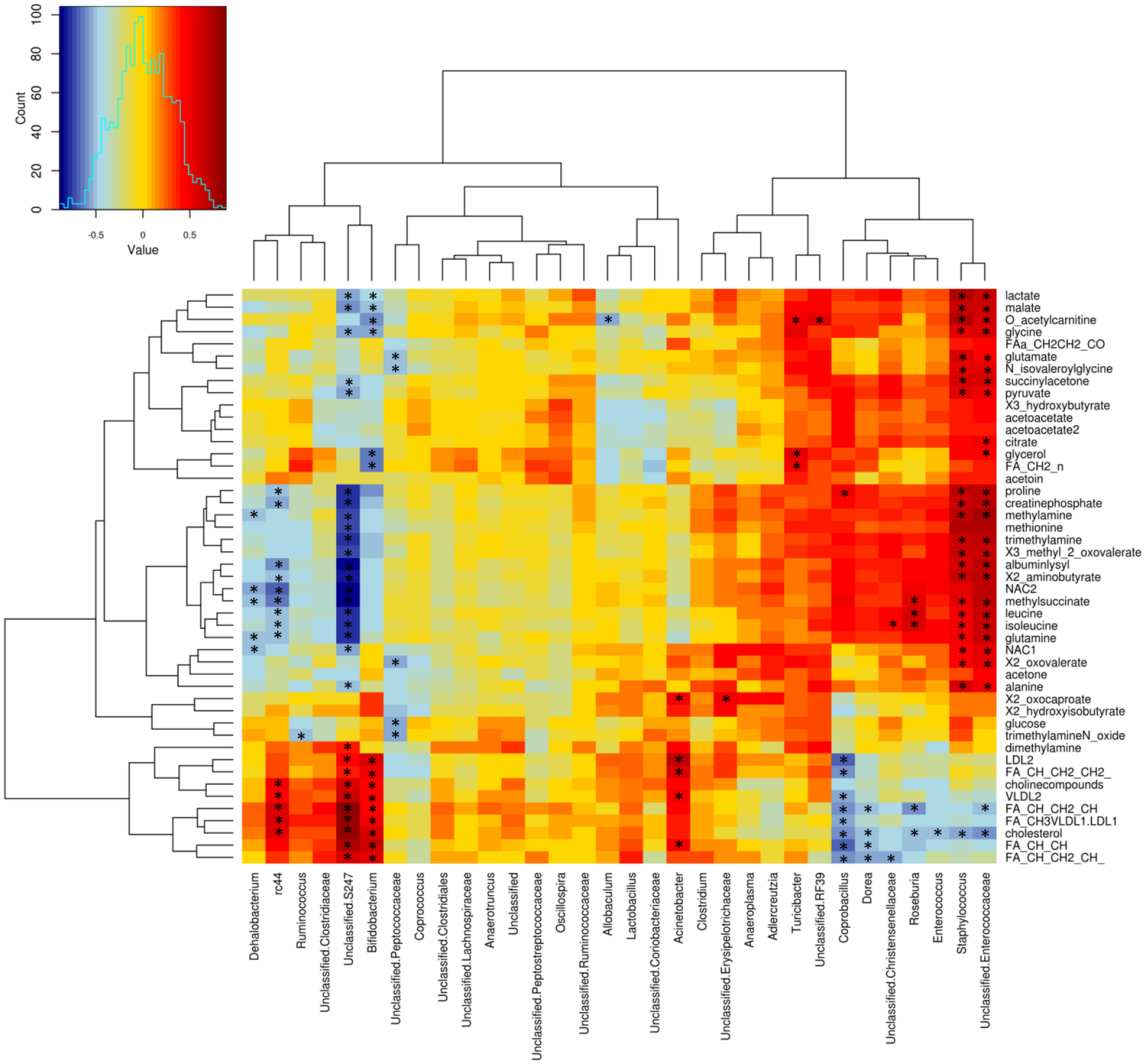
Associations between colon microbiome and serum metabolome. Heatmap shows associations between metabolite profile and relative abundance of specific bacterial families and genera in ND and 3xtg groups. Red to blue scale: positive to negative associations. Pearson’s correlations were employed in agreement with data distribution, verified by Shapiro-Wilk test. *p < 0.05.

Heatmap generated to examine correlations between colon microbiota and serum metabolome in ND and 3xtg mice (Fig. 7) showed three main clusters: I) lactate, malate, acetylcarnitine, glutamate, succinylacetone, glycerol, citrate, etc; II) proline, methylalanine, methionine, creatine phosphate, leucine, isoleucine, glutamine, alanine, methylsuccinate, etc; III) cholesterol, lipoproteins and fatty acids. *Bifidobacterium* and *Unclassified s24.7* levels, which were shown to be defective 3xtg mice, were related to high lactate, malate and aminoacid levels, and to low cholesterol, low density lipoprotein (LDL) and fatty acid amounts. *Coprobacillus*, *Dorea* and other bacterial strains were also negatively related to cholesterol and fatty acid levels. Abundance of *rc4.4*, which was shown to be deficient in 3xtg mice, was associated negatively with circulating aminoacids and positively with cholesterol and fatty acids levels. Both *Unclassified s24.7* and *rc4.4* were negatively correlated with the amount of circulating choline compounds and inflammatory marker GlycA. Conversely, *Staphylococcus* and *Unclassified Enterococcus* (shown to be overabundant in 3xtg) were positively associated with lactate, malate, acetylcarnitine, succinylacetone, pyruvate and aminoacid levels. Overall, bacteria correlating with an adverse metabolic profile, as defined by 3xtg characteristics, were primarily *Enterococcaceae*, *Staphylococcus*, followed by *Roseburia*, *Coprobacillus*, *Dorea*, *Enterococcus*, *Christenellaceae*. The ones relating to the reverse healthier profile were *s24.7*, *rc4.4*, *Bifidobacterium*, *Dehalobacterium*, *Peptococcaceae*, *Acinetobacter*.

### Microbiome and brain associations

**Figure 8:**
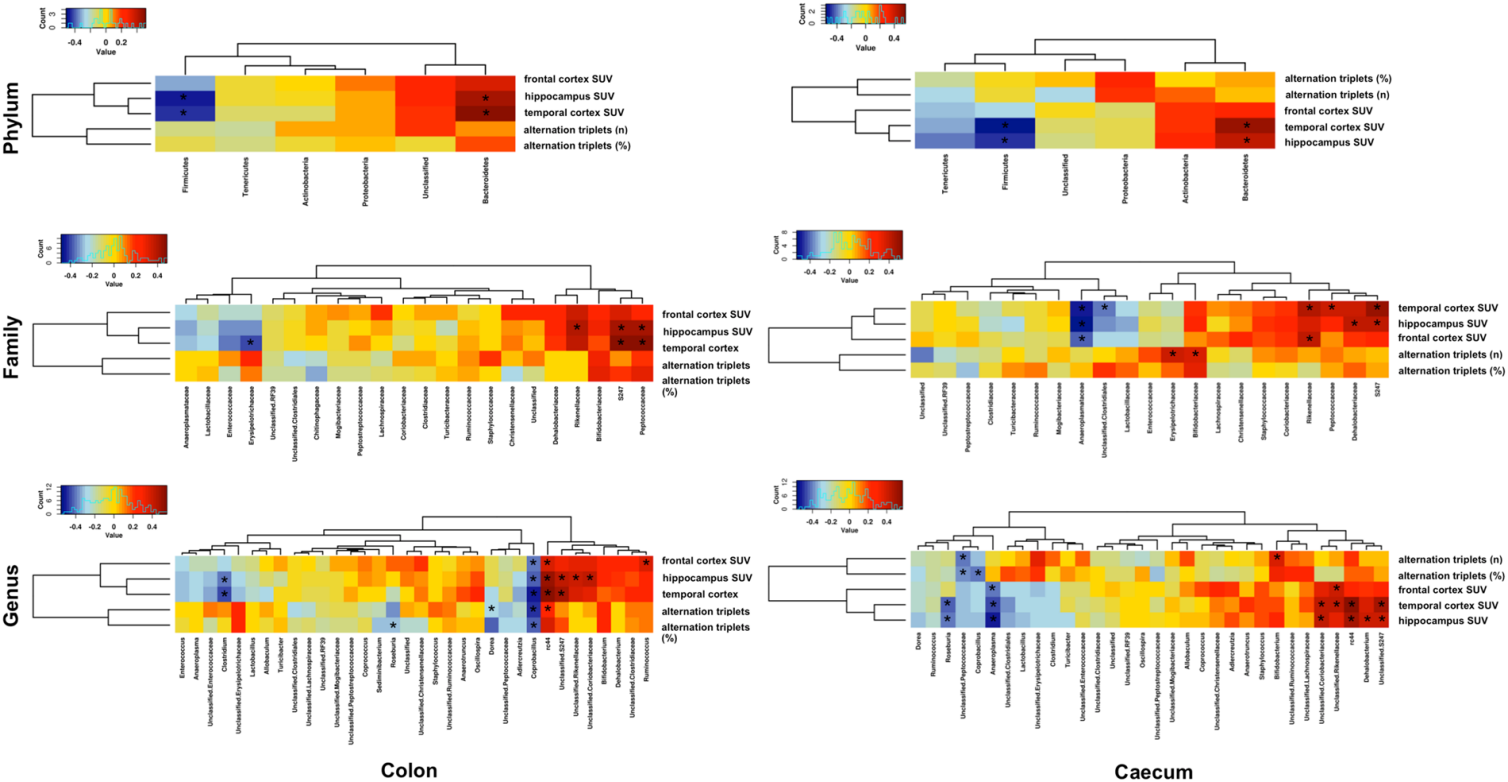
Associations between gut microbiome and cerebral parameters. Heatmap generated from Spearman correlation analysis shows associations between brain parameters (cognitive function and glucose metabolism) and relative abundance of specific bacterial phyla, families and genera across groups, in caecum and colon. Red to blue scale: positive to negative associations. Spearman’s correlations were employed in agreement with data distribution, verified by Shapiro-Wilk test. *p < 0.05. *p < 0.05.

Heatmap generated to examine correlations between caecum-colon microbiota and cerebral parameters (Alternation triplets, n and %, and glucose metabolism) across groups (Fig. 8) showed that cerebral hypometabolism was associated with higher abundance of Firmicutes phylum, *Anaeroplasmataceae* and *Erysipelotrichaceae* families, and *Coprobacillus*, *Clostridium*, *Anaeroplasma* and *Roseburia* genera, and lower abundance of Bacteroidetes phylum, *Peptococcaceae*, *Rikenellaceae*, *Dehalobacteriaceae* and *s24.7* families, and *rc4.4*, *Dehalobacterium*, *Unclassified Coriobacteriacea*, *Unclassified Rikenellaceae*, *Unclassified s24.7*.

Furthermore, we observed that higher abundance of *Bifidobacteriaceae* and *Erysipelotrichaceae* families, and *Bifidobacterium* and *rc4.4* genera were positively correlated with cognitive function, whereas negative associations occurred with *Coprobacillus*, *Dorea*, *Roseburia*, and *Unclassified Peptococcaceae*.

## Discussion

The emerging biology of gut-brain crosstalk has revealed the existence of a complex bidirectional system^25^, in which neurodegenerative processes affecting the brain also occur in the gut^5,6^, and vice versa, factors primarily influencing the gut microbiota, e.g. high-fat diets, increase the risk of neurodegeneration. We addressed these interrelations by comparing normal and high-fat feeding in control mice and in a transgenic mouse model of neurodegenerative disease. This model was studied prior to the development of full-blown cognitive deficits, which we have shown to occur later in life^26^. In the current disease stage, our 3xtg mice showed the typical cerebral hypometabolism that precedes dementia in humans^27^. Several novel results emerged from our study.

The most salient finding was the dramatic rightward and clear-cut shift occurring in the overall composition of the microbiota in 3xtg compared to ND mice, and defining a global microbiota pattern which enabled to clearly identify subjects that are going to be demented. Another specific trait of 3xtg mice was the loss of differences in microbial diversity between caecum and colon, as opposed to control mice, in which the caecum showed superior diversity than the colon, due to higher amounts of Firmicutes and lower Bacteroidetes at phylum level, and higher *Lachnospiraceae* and lower *S24.7* at family level. Bacterial richness was also defective in 3xtg mice, as documented by Chao1 indices. In normal gut physiology, the caecum and colon reflect very different functions, and a greater diversity in their microbial composition - as observed in our healthy mice - is therefore expected. The lack of this trait in our 3xtg mice may indicate that these intestinal segments have lost their distinctive milieu. In fact, recent reports document that neurodegeneration involves intestinal walls (beyond brain), leading to functional abnormalities in 3xtg mice^5,6^. This may affect microbial composition and function. Notably, 3xtg mice were characterized by an elevation in Firmicutes compared to Bacteroidetes abundance in both gut tract, and these traits were associated with cerebral hypometabolism. Most important, specific families were exclusively represented in the 3xtg model, and absent in ND mice. These were *Turicibacteraceae* (colon), *Christensenellaceae* (caecum) and *Anaeroplasmataceae* (both colon and caecum). Conversely, *Bifidobacteriaceae* were absent in 3xtg mice, while being well represented in the control ND model (colon and caecum), in agreement with their positive association with cognitive function. In the colon, 3xtg mice also showed higher amounts of *Enterococcaceae*. In the caecum, lower abundance of *S24.7* and *rc4.4*, and higher amounts of *Ruminoccaceae* and *Roseburia* occurred. Some, but not all of these bacterial alterations, along with the reduction in bacterial diversity and richness have been described in dysmetabolic, low-grade inflammatory and inflammatory bowel states^28–31^, in agreement the high serum levels of GlycA (marker of inflammation) observed in our 3xtg mice. Our data lend support to the concept that abundance differences in very selected bacterial families are specifically related to a high-risk to develop dementia in our mouse model, and warrant studies to examine direct cause-effect relationships.

We explored serum and faecal metabolomes, and their correlations with bacteria. Notably, our PCA analysis explained 46% of changes in serum metabolites occurring in 3xtg compared to ND mice, indicating a strong and selective relationship between disease and metabolome. In fact, subgroup analyses showed well-defined serum and faecal metabolite clusters in 3xtg versus control mice. Also important was that these metabolomics alterations were significantly correlated with the prevalence of *Enterococcaceae*, *Coprobacillus*, *Staphylococcus*, *Roseburia* and *Dorea*, belonging to the Firmicutes phylum, mostly in the *Clostridia* and *Lachnospiraceae* class and families, and with deficiencies in *S24.7*, *rc4.4*, *Bifidobacterium*, *Dehalobacterium*. Coherently, associations between most of these taxa and cerebral metabolism and/or cognitive function were also detected. One striking finding in faecal metabolites was the elevated presence of amino acids ornithine and thyrosine. Ornithine provides cerebral protection against ammonia, whereas thyrosine has neuroexcitatory properties, and *Clostridia* modulate amino acid metabolism. The pathophysiological significance of this finding in the context of neurodegeneration deserves further investigation. Serum metabolomics patterns seen in 3xtg mice were typical of neurodegenerative diseases. First, reductions in unsaturated fatty acids, LDL and VLDL, as seen in 3xtg mice, compromise the important contribution of lipids to neuronal cell structure (50% of neuronal membranes), energy storage and signal transduction, and relate to the development of AD^16–18^. Second, the brain is an avid consumer of glucose, and our 3xtg mice showed a gross impairment in brain glucose uptake. The accompanying increase in ketone bodies, i.e. β-hydroxybutyrate, acetoacetate, and acetone may serve as protective alternative fuel during brain glucose starvation, as suggested in humans with mild-cognitive impairment (MCI)^32^. The impairment in brain glucose metabolism has been implicated in the pathogenesis of hyperglycemia and hyperlactatemia in patients with MCI and AD^32,33^, and these substrate excesses were found in our 3xtg mice. Lactate is a modulator of cerebral Aβ production, and its levels are abnormal in brain and cerebrospinal fluid (CSF) of AD subjects^34,35^. Third, remarkable alterations were found in amino acid metabolism in 3xtg mice, consisting in high levels of methionine (precursor of homocysteine), leucine (activator of the mammalian target of rapamycin, mTOR, signalling^36^) and isoleucine (relating to insulin resistance with other branched-chain amino acids^37^), alanine and glutamate (important in neurotransmission). These pathways have been implicated in the pathogenesis of cerebrovascular disease and dementia^38^, β-amyloid brain accumulation^39^, enhanced neurofibrillar tangle formation^40^, and glutamatergic dysregulation^21^. We also report elevated TMA, TMAO levels (regulating tau aggregation^41^), and choline deficiency (precursor of acetylcholine, a cholinergic neurotransmitter implicated in memory and AD^42^), in 3xtg mice. Altogether, these results suggest that metabolite changes known to compromise brain structure and function occur in 3xtg mice, prior to the development of overt dementia, and correlate with the abundance of selected gut bacteria, revealing potential mechanisms underlying the complex interaction linking gut and neurodegenerative disease.

High-fat diets have a profound impact on gut microbiota^9^. Extending previous observations, we found that a HFD modifies bacterial composition in both colon and caecum, also leading to lower richness compared to ND. In both caecum and colon, HFD mice showed high relative abundances of Firmicutes than Bacteroidetes at phylum levels, *Rikenellaceae*, *Lachnospiraceae*, *Enterococcaceae* and *S24.7* at family level, as well as increased amount of faecal ribose. Higher post-prandial colonic motility has been described in response to HFD consumption, and ribose affects gut activity^43,44^. Elevations in *Clostridium* and *Staphylococcus* were also observed in the caecum. HFD mice were depleted in protective *Bifidobacteriaceae* and *Lactobacillaceae* (*Bifidobacterium* and *Lactobacillus* at genus level), which maintain mucosal barrier integrity and constrain endotoxin formation, preventing inflammation and metabolic complications^45,46^. Coherently with this, GlycA levels were high in our HFD mice.

We and others have shown that a HFD promotes cognitive impairment in both humans and mice, and that the acceleration of cognitive decline is particularly evident in older 3xtg-HFD mice^26^. In this study, compared to HFD or 3xtg alone, summative outcomes were seen in selected microbial families and genera in 3xtg-HFD mice, resulting in severe depletion of *S24.7*, *Peptococcaceae*, *Dehalobacteriaceae* families, and *rc4.4* genus, and excess of *Clostridium* genus abundance. In the metabolome of HFD and 3xtg mice, most changes occurred in a similar direction, leading to strong additive effects in 3xtg-HFD mice, compared to HFD and 3xtg alone. One important distinctive feature was also noted, as 3-hydroxybutyrate and acetoacetate were high in 3xtg and low in HFD, compared to ND mice. Ketones are considered protective in Alzheimer’s brains, as they may compensate for cerebral glucose hypometabolism^47,48^. Therefore, the ketone deficiency caused by HFD in our 3xtg-HFD mice can be an important mechanism whereby HFD accelerates progression of cognitive impairment in 3xtg mice^26^.

The study has some limitations. It involved male mice to limit data variability, considering that males seem more vulnerable than females to the impact of HFD-induced obesity (i.e. a target risk factor for dementia), on weight gain, metabolic alterations, deficits of learning, and hippocampal synaptic plasticity^49^. However, it would be relevant to extend findings to females, since the number of women with obesity and/or dementia is also high. Furthermore, the study was conducted in mice, and therefore its translational significance remains to be examined.

In conclusion, our study shows that high-fat feeding and genetic predisposition to neurodegenerative disease share abnormalities in the resulting microbiome and metabolome, which are additive and seem unfavourable to brain health. Most important, distinctive signatures, including selected microbial families and metabolic changes, and the lack of bacterial diversity between colon and caecum were exclusively found in association with 3xtg mice, and coherent associations were identified between microbiota changes and cognitive reductions or cerebral hypometabolism. Our findings have important implications, as an excellent discrimination was shown in microbial-metabolome profiles between groups, and 3xtg mice were pre-demented, suggesting that the above clear-cut microbiome-metabolome deviations occur early in the course of neurodegenerative disease.

## Methods

### Animal model and study design

The study involved n = 18 B6129SF2/J (stock no:101045, The Jackson Laboratory, Bar Harbor, Maine) and n = 15 triple transgenic (3xtg) male mice (B6;129-Psen^1tm1Mpm^Tg[APPSwe, tauP301L]1Lfa/Mmjax stock no:004807, MMRRC034830, The Jackson Laboratory), randomly stratified as follow: I) normal diet-fed mice (ND, B6129SF2J, n = 9, 11% kcals from fat, Mucedola, Italy); II) high-fat diet-fed mice (HFD, B6129SF2J, n = 9, 58% kcals from fat, Mucedola); III) ND-fed 3xtg mice (3xtg, n = 8) and IV) HFD-fed 3xtg mice (3xtg-HFD, n = 7). The slight discrepancy in group sizes was due to higher 3xtg mortality. Animals were housed in standard cages (n = 4–5 mice/cage) under controlled conditions (12-hour light/12-hour dark cycles, 22 °C), with *ad libitum* access to food and water. Veterinary and animal care staffs were responsible for the monitoring of animal welfare and health for all the duration of the study. Diet was administered from 2 to 8 months of age, when body weight, cognitive performance and brain glucose metabolism were assessed. Then, animals were euthanized by an anaesthetic overdose, and blood and faecal samples (caecum and colon) collected for analyses of serum and faecal metabolome, and gut microbiota. The experimental protocol was notified to the Ministry of Health (Dept. of Public Veterinary Health) in accordance with the D.L.116/92 implementation of directive EEC 609/86 regarding the protection of animals used for experimental and other scientific purposes, and adheres to standards articulated in Reporting of *In Vivo* Experiments (ARRIVE) guidelines.

### Cognitive analysis by Y-maze test

Cognitive performance was measured by a spontaneous alternation test (Y-maze, Panlab, Harvard Apparatus, Spain). Each subjects was allowed to freely move through the maze for 8 minutes, whereas a visual automatic tracking system (Panlab) recorded the number of alternation triplets explored.

### Brain glucose metabolism by [^18^F]FDG PET/CT

On the experimental day, each animal was transferred to the imaging facility. Anaesthesia was induced and maintained in overnight-fasted mice by ∼2% (v/v) isofluorane inhalation (IsoFlo^**®**^, Abbott Laboratories, IL, USA), a standard method for rodent based on minimal handling and safety, and breath frequency and body temperature were monitored. Then, a 60-minute dynamic PET scan was acquired after i.p. ^18^F]FDG injection, by an IRIS PET/CT small-animal tomograph (Inviscan SAS, France). Volumes of interest were manually drawn on PET/CT images by AMIDE software (AMIDE-bin 1.0.5) in the hippocampus, frontal and temporal cortex. Regional [^18^F]FDG uptake was expressed as standardized uptake value (SUV), i.e. the ratio between tissue activity (kBq/ml) at 60 min post-injection, and weight-normalized injected dose (kBq/g).

### Serum and faecal metabolites by 1H-NMR Spectroscopy

Blood samples (n = 33) were centrifuged (2200 RCF, 10 min) to obtain serum. Faecal samples (n = 27, caecum; n = 28, colon) were suspended in Milliq water (100 µL) and centrifuged (14800 rpm, 5 minutes) for 3 consecutive times to obtain the extract. Extracts (20 μl) supplemented with D_2_O (2 μl) were transferred into 1-mm NMR tubes. 1H-NMR spectra (8000 Hz width) were recorded in a Bruker Avance DRX 600 spectrometer (Valencia, Spain), equipped with a triple resonance 1 H/13 C/31 P probe. Samples were measured at 310 K and a single-pulse presaturation experiment was acquired in all samples. The water signal was saturated with weak irradiation during the recycle delay. Data were Fourier transformed after the free induction decay was multiplied by a 0.3 Hz exponential line-broadening function. Spectra were processed using MestReNova 8.1 (Mestrelab Research S.L., Spain) and transferred to MATLAB (MathWorks, 2012) using in-house scripts for data analysis. The chemical shift region including resonances 0.50–4.70 ppm (the aliphatic region) and 5.20–10.00 ppm (the aromatic region) was investigated. Metabolite spin systems and resonances were identified by literature data and Chenomx resonances database (Chenomx NMR 7.6). Spectra were normalized to total aliphatic spectral area to eliminate differences in metabolite total concentration, binned into 0.01 ppm buckets and then subjected to mean-centering before multivariate analysis. Signals belonging to selected metabolites were integrated and quantified using semi-automated in-house MATLAB peak-fitting routines. Final metabolite levels were calculated in arbitrary units as peak area normalized to total spectral area.

### NMR profile and statistical analyses

Metabolomics data analysis was performed using in-house MATLAB scripts and PLS_Toolbox (Eigenvector Research). PCA was applied to NMR spectra data sets, to find low-dimensional embeddings of multivariate data. PCs were chosen to explain at least 70% of the variance. The loading plots of the corresponding PC were used to detect the position of most discriminative variables in the NMR spectra. To maximize the separation between samples, PLS-DA was applied. Permutation test was performed to check overfitting of the PLS-DA models. The multivariate chemometric models were cross-validated with 10-fold Venetian blind cross-validation. In each run, 10% of data were left out of the training and used to test the model. Spectral regions with high variable importance in projections (VIP) coefficients obtained during PLS-DA are more important in providing class separation during analysis, while those with small VIP coefficients provide little contribution to classification. P-values ≤ 0.05 were regarded as statistically significant.

### Faecal microbiome by 16S rDNA gene sequencing

Total DNA was isolated from faecal samples (n = 27, caecum; n = 28, colon) by MasterPure Complete DNA&RNA Purification Kit (Epicentre, Illumina, WI, USA), with some modifications, as previously described^50^. Isolated DNA concentrations were measured using a Qubit® 2.0 Fluorometer (Life Technology, CA, USA) and normalized to 10 ng/μL. The V3-V4 region of 16 S rDNA gene was amplified by PCR using Illumina adapter overhang nucleotide sequences following Illumina protocols. The multiplexing step was performed using Nextera XT Index Kit (Illumina, CA, USA). PCR product was checked with a Bioanalyzer DNA 1000 chip (Agilent Technologies, CA, USA) and libraries were sequenced using a 2 × 300 pb paired-end run (MiSeq Reagent kit v3) on a MiSeq-Illumina platform (FISABIO sequencing service, Valencia, Spain) according to manufacturer’s instructions (Illumina). Reagents employed for DNA extraction and PCR amplification were also sequenced as controls.

### Bioinformatics and statistical analysis

Quality assessment was performed by prinseq-lite program (min_length:50; trim_qual_right:20; trim_qual_type:mean; trim_qual_window:20^51^). R1 and R2 from sequencing where joined using fastq-join from ea-tools suite^52^. Data were obtained using an ad-hoc pipeline written in RStatistics environment^53^ and data processing was performed by QIIME pipeline (version 1.9.0)^54^. Chimeric sequences and sequences that could not be aligned were removed. The clustered sequences were utilized to construct OTUs tables (97% identity), then classified into phylum, family, genus taxonomic levels based on the Greengenes database. Sequences, that could not be classified, or classified as Cyanobacteria and Chloroplasts (representing ingested plant material), were removed. Alpha diversity indices (Chao1 and Shannon), UniFrac beta diversity, and PERMANOVA were used to test significance. ANOSIM test was employed for group-comparison in microbiota communities. Calypso software version 7.36 (http://cgenome.net/calypso/) was used with total sum normalization (TSS) for the statistical analysis, and also Cumulative Sum Scaling normalisation (CSS) for multivariate tests (RDA). Venn diagram for shared phylotypes was generated with the same software. LEfSe was used to detect unique biomarkers (LDA score >3.0) in relative abundance of bacterial taxonomy^55^. P-values ≤ 0.05 were regarded as statistically significant.

### Data availability

The datasets generated and analysed during the current study are not publicly available, but will be available from the corresponding author on reasonable request.

## Electronic supplementary material

Supplementary Tables and Figures
