# Supplementary material for: Microbiome-metabolome signatures in mice genetically prone to develop dementia, fed a normal or fatty diet
Source: Sci Rep. 2018 Mar 20;8:4907. doi: 10.1038/s41598-018-23261-1 (PMC5861049; doi:10.1038/s41598-018-23261-1)
Supplement: Supplementary file 1 — Supplementary Tables and Figures [file 41598_2018_23261_MOESM1_ESM.doc]

**Microbiome-metabolome signatures in mice genetically prone to develop dementia, fed a normal or fatty diet**

Elena Sanguinetti^+^, Maria Carmen Collado^+^, Vannina G. Marrachelli, Daniel Monleon, Marta Selma-Royo, Mercedes M. Pardo-Tendero, Silvia Burchielli, Patricia Iozzo^*^

^+^These authors contributed equally to this work

*Corresponding author

**Supplementary tables and figures**

**Supplementary Table S1. Serum metabolites.**

|  |  |  |  |  |  |  | | **ND** | | **HFD** | | **3xtg** | | **3xtg-HFD** | |  |
| --- | --- | --- | --- | --- | --- | --- | --- | --- | --- | --- | --- | --- | --- | --- | --- | --- |
|  |  |  |  |  |  | **Region** | **Metabolite** | **mean** | **SD** | **mean** | **SD** | **mean** | **SD** | **mean** | **SD** | **p** |
|  |  |  |  |  |  | 1 | cholesterol | 0.73 | 0.01 | 0.72 | 0.01 | 0.70 | 0.01 | 0.70 | 0.02 | 6.99E-06 |
|  |  |  |  |  |  | 2 | FA -CH3 (VLDL1/LDL1) | 5.76 | 0.70 | 5.54 | 0.71 | 4.31 | 0.69 | 4.39 | 0.90 | 3.46E-04 |
|  |  |  |  |  |  | 3 | 2-oxocaproate | 0.84 | 0.19 | 0.98 | 0.32 | 0.87 | 0.09 | 0.94 | 0.20 | 5.58E-01 |
|  |  |  |  |  |  | 6 | leucine | 0.86 | 0.10 | 0.90 | 0.06 | 1.06 | 0.09 | 1.05 | 0.07 | 1.28E-05 |
|  |  |  |  |  |  | 7 | isoleucine | 0.67 | 0.06 | 0.71 | 0.05 | 0.80 | 0.08 | 0.82 | 0.06 | 9.14E-05 |
|  |  |  |  |  |  | 9 | FA (-CH2-)n | 5.15 | 1.30 | 7.06 | 1.71 | 6.49 | 1.24 | 11.14 | 9.83 | 9.62E-02 |
|  |  |  |  |  |  | 11 | VLDL2 | 5.11 | 0.75 | 4.88 | 0.74 | 3.92 | 0.81 | 3.91 | 1.20 | 1.30E-02 |
|  |  |  |  |  |  | 12 | LDL2 | 7.60 | 2.18 | 8.10 | 3.05 | 5.68 | 1.53 | 6.68 | 3.28 | 2.56E-01 |
|  |  |  |  |  |  | 14 | 2-hydroxyisobutyrate | 0.29 | 0.06 | 0.31 | 0.06 | 0.29 | 0.03 | 0.31 | 0.07 | 8.27E-01 |
|  |  |  |  |  |  | 16 | alanine | 1.08 | 0.14 | 1.16 | 0.09 | 1.22 | 0.09 | 1.43 | 0.34 | 5.21E-03 |
|  |  |  |  |  |  | 17 | 2-oxovalerate | 1.16 | 0.16 | 1.26 | 0.16 | 1.30 | 0.07 | 1.39 | 0.17 | 3.21E-02 |
|  |  |  |  |  |  | 19 | 2-aminobutyrate | 0.58 | 0.08 | 0.65 | 0.08 | 0.79 | 0.09 | 0.83 | 0.12 | 8.97E-06 |
|  |  |  |  |  |  | 22 | FA =CH-CH2-CH2- | 2.95 | 0.47 | 3.07 | 0.54 | 2.63 | 0.32 | 2.74 | 0.35 | 1.78E-01 |
|  |  |  |  |  |  | 23 | GlycA1 | 1.00 | 0.13 | 1.11 | 0.12 | 1.15 | 0.05 | 1.22 | 0.13 | 3.99E-03 |
|  |  |  |  |  |  | 24 | GlycA2 | 0.55 | 0.08 | 0.65 | 0.08 | 0.82 | 0.11 | 0.83 | 0.11 | 1.26E-06 |
|  |  |  |  |  |  | 25 | methylsuccinate | 0.09 | 0.01 | 0.10 | 0.01 | 0.13 | 0.02 | 0.13 | 0.02 | 5.62E-06 |
|  |  |  |  |  |  | 27 | N-isovaleroylglycine | 0.24 | 0.03 | 0.25 | 0.01 | 0.27 | 0.02 | 0.27 | 0.02 | 5.02E-02 |
|  |  |  |  |  |  | 28 | FA a-CH2 (CH2-CO) | 1.28 | 0.29 | 1.31 | 0.33 | 1.44 | 0.34 | 1.27 | 0.26 | 6.89E-01 |
|  |  |  |  |  |  | 29 | acetoin | 0.35 | 0.13 | 0.34 | 0.16 | 0.38 | 0.21 | 0.23 | 0.07 | 2.58E-01 |
|  |  |  |  |  |  | 30 | acetone | 0.61 | 0.15 | 0.63 | 0.18 | 0.69 | 0.12 | 0.70 | 0.19 | 5.71E-01 |
|  |  |  |  |  |  | 31 | succinylacetone | 0.11 | 0.02 | 0.12 | 0.01 | 0.14 | 0.02 | 0.14 | 0.01 | 2.08E-03 |
|  |  |  |  |  |  | 32 | acetoacetate | 0.33 | 0.14 | 0.22 | 0.06 | 0.45 | 0.30 | 0.22 | 0.06 | 3.33E-02 |
|  |  |  |  |  |  | 34 | glutamate | 9.93 | 1.38 | 10.30 | 0.75 | 11.21 | 1.04 | 11.81 | 1.10 | 7.24E-03 |
|  |  |  |  |  |  | 35 | pyruvate | 0.13 | 0.03 | 0.13 | 0.02 | 0.17 | 0.03 | 0.18 | 0.03 | 2.23E-03 |
|  |  |  |  |  |  | 36 | 3-hydroxybutyrate | 0.43 | 0.18 | 0.32 | 0.07 | 0.55 | 0.30 | 0.34 | 0.06 | 6.43E-02 |
|  |  |  |  |  |  | 38 | glutamine | 0.33 | 0.05 | 0.36 | 0.05 | 0.47 | 0.09 | 0.43 | 0.08 | 1.36E-03 |
|  |  |  |  |  |  | 39 | citrate | 3.62 | 0.89 | 3.25 | 0.31 | 4.66 | 1.56 | 3.73 | 0.45 | 2.99E-02 |
|  |  |  |  |  |  | 40 | methylamine | 0.01 | 0.00 | 0.01 | 0.00 | 0.01 | 0.00 | 0.01 | 0.00 | 3.14E-05 |
|  |  |  |  |  |  | 42 | methionine | 0.12 | 0.02 | 0.15 | 0.02 | 0.18 | 0.02 | 0.20 | 0.04 | 8.68E-06 |
|  |  |  |  |  |  | 43 | malate | 0.04 | 0.01 | 0.05 | 0.00 | 0.06 | 0.00 | 0.07 | 0.01 | 6.09E-06 |
|  |  |  |  |  |  | 46 | dimethylamine | 0.12 | 0.02 | 0.11 | 0.01 | 0.11 | 0.01 | 0.11 | 0.02 | 8.02E-01 |
|  |  |  |  |  |  | 47 | FA =CH-CH2-CH= | 0.98 | 0.11 | 0.87 | 0.18 | 0.70 | 0.20 | 0.58 | 0.14 | 1.52E-04 |
|  |  |  |  |  |  | 48 | FA =CH-CH2-CH= | 0.71 | 0.10 | 0.67 | 0.15 | 0.59 | 0.12 | 0.49 | 0.08 | 4.87E-03 |
|  |  |  |  |  |  | 49 | trimethylamine | 0.03 | 0.01 | 0.03 | 0.01 | 0.04 | 0.00 | 0.04 | 0.01 | 7.06E-06 |
|  |  |  |  |  |  | 50 | 3-methyl-2-oxovalerate | 0.38 | 0.07 | 0.43 | 0.09 | 0.54 | 0.06 | 0.56 | 0.09 | 8.37E-05 |
|  |  |  |  |  |  | 51 | albumin lysyl | 0.24 | 0.04 | 0.30 | 0.05 | 0.38 | 0.05 | 0.41 | 0.07 | 6.84E-07 |
|  |  |  |  |  |  | 52 | creatine phosphate | 0.09 | 0.01 | 0.11 | 0.01 | 0.14 | 0.02 | 0.17 | 0.05 | 2.83E-05 |
|  |  |  |  |  |  | 55 | O-acetylcarnitine | 0.05 | 0.01 | 0.06 | 0.01 | 0.07 | 0.01 | 0.09 | 0.02 | 2.81E-06 |
|  |  |  |  |  |  | 56 | choline compounds | 1.70 | 0.32 | 1.79 | 0.29 | 1.34 | 0.22 | 1.45 | 0.40 | 2.01E-02 |
|  |  |  |  |  |  | 58 | trimethylamine N-oxide | 0.21 | 0.14 | 0.23 | 0.12 | 0.26 | 0.07 | 0.38 | 0.15 | 5.80E-02 |
|  |  |  |  |  |  | 60 | proline | 0.10 | 0.02 | 0.13 | 0.02 | 0.18 | 0.03 | 0.21 | 0.06 | 4.02E-06 |
|  |  |  |  |  |  | 63 | glucose | 1.10 | 0.83 | 1.25 | 0.79 | 1.53 | 0.49 | 2.51 | 1.39 | 2.28E-02 |
|  |  |  |  |  |  | 64 | glycine | 0.20 | 0.09 | 0.14 | 0.03 | 0.28 | 0.17 | 0.18 | 0.08 | 4.83E-02 |
|  |  |  |  |  |  | 66 | glicerol | 0.15 | 0.03 | 0.22 | 0.05 | 0.22 | 0.04 | 0.39 | 0.31 | 2.30E-02 |
|  |  |  |  |  |  | 73 | lactate | 0.87 | 0.18 | 1.23 | 0.25 | 1.10 | 0.15 | 1.83 | 1.24 | 2.65E-02 |
|  |  |  |  |  |  | 77 | FA-CH=CH | 0.28 | 0.09 | 0.43 | 0.14 | 0.43 | 0.10 | 0.58 | 0.22 | 2.16E-03 |

| **Supplementary Table S2.** Faecal metabolites. | | | | | | | | | | | | | | | | | | |
| --- | --- | --- | --- | --- | --- | --- | --- | --- | --- | --- | --- | --- | --- | --- | --- | --- | --- | --- |
|  |  | **ND**  **colon** | | **ND**  **caecum** | | **HFD**  **colon** | | **HFD**  **caecum** | | **3xtg-HFD**  **colon** | | **3xtg-HFD**  **caecum** | | **3xtg**  **colon** | | **3xtg-HFD**  **caecum** | |  |
| **Region** | **Metabolites** | **Mean** | **SD** | **Mean** | **SD** | **Mean** | **SD** | **Mean** | **SD** | **Mean** | **SD** | **Mean** | **SD** | **Mean** | **SD** | **Mean** | **SD** | **p** |
| 1 | FA | 17.94 | 7.08 | 13.08 | 11.76 | 29.65 | 5.38 | 27.21 | 5.82 | 30.71 | 15.01 | 23.73 | 11.96 | 24.50 | 10.35 | 24.46 | 8.91 | 0.03 |
| 3 | leucine | 57.27 | 14.64 | 56.74 | 9.16 | 55.88 | 11.36 | 56.04 | 6.42 | 52.62 | 5.86 | 53.48 | 12.78 | 61.30 | 5.10 | 61.69 | 21.48 | 0.90 |
| 4 | 2-aminobutyratea | 11.19 | 3.17 | 10.84 | 2.36 | 13.74 | 2.30 | 12.43 | 2.25 | 11.74 | 1.70 | 10.81 | 1.35 | 12.05 | 1.55 | 14.89 | 2.28 | 0.05 |
| 5 | isoleucine | 18.37 | 6.07 | 19.73 | 3.06 | 11.75 | 5.52 | 16.13 | 3.78 | 12.13 | 4.20 | 16.38 | 5.34 | 14.83 | 3.10 | 18.44 | 7.28 | 0.05 |
| 6 | valine | 24.17 | 6.21 | 25.21 | 5.25 | 19.96 | 5.49 | 24.79 | 2.48 | 22.13 | 4.13 | 23.82 | 4.49 | 22.66 | 3.14 | 26.63 | 6.82 | 0.48 |
| 7 | Isobutyrate+propionate | 5.38 | 3.15 | 6.44 | 3.09 | 3.03 | 0.98 | 6.08 | 0.80 | 4.33 | 2.82 | 6.59 | 3.06 | 2.87 | 1.88 | 6.33 | 1.71 | 0.06 |
| 8 | 2-methylglutarate | 0.60 | 0.64 | 0.36 | 0.23 | 0.18 | 0.29 | 0.60 | 0.47 | 0.28 | 0.56 | 0.87 | 0.63 | 0.35 | 0.56 | 0.61 | 0.62 | 0.38 |
| 9 | 3-methyl-2-oxovalerate | 2.28 | 1.61 | 1.51 | 1.00 | 2.05 | 1.59 | 3.28 | 1.07 | 2.40 | 2.34 | 3.03 | 1.63 | 1.74 | 1.81 | 2.25 | 1.60 | 0.59 |
| 10 | propylene glycol | 1.99 | 1.37 | 0.91 | 0.76 | 1.00 | 0.84 | 0.93 | 0.80 | 0.77 | 1.15 | 1.29 | 1.04 | 0.59 | 0.93 | 1.22 | 1.44 | 0.33 |
| 11 | ethanol | 5.95 | 2.27 | 3.65 | 1.08 | 3.95 | 1.26 | 2.99 | 1.20 | 3.40 | 1.96 | 3.24 | 1.30 | 3.37 | 1.28 | 3.38 | 2.83 | 0.05 |
| 12 | 3-aminoisobutyrate | 9.20 | 3.57 | 5.50 | 2.19 | 5.70 | 1.99 | 4.16 | 2.17 | 5.40 | 2.37 | 4.10 | 1.50 | 4.51 | 1.68 | 4.23 | 3.39 | 0.01 |
| 13 | 3-hydroxybutyrate | 2.65 | 1.86 | 2.07 | 1.23 | 2.69 | 1.16 | 1.31 | 0.85 | 1.63 | 1.19 | 1.30 | 0.52 | 2.46 | 0.94 | 1.41 | 1.35 | 0.26 |
| 14 | lactate + threonine | 46.78 | 25.19 | 39.49 | 15.35 | 55.76 | 22.95 | 31.68 | 2.21 | 39.16 | 11.71 | 30.80 | 3.85 | 34.43 | 6.41 | 29.04 | 4.22 | 0.09 |
| 16 | alanine | 30.98 | 6.83 | 35.49 | 5.69 | 25.13 | 6.51 | 30.50 | 5.63 | 25.39 | 5.69 | 30.00 | 9.07 | 27.85 | 3.75 | 31.86 | 9.51 | 0.12 |
| 21 | glutarate | 4.60 | 3.60 | 3.35 | 3.41 | 8.15 | 4.87 | 8.79 | 3.38 | 8.46 | 4.29 | 7.21 | 2.74 | 7.21 | 2.05 | 6.59 | 5.16 | 0.11 |
| 23 | acetate | 25.42 | 16.43 | 27.79 | 14.24 | 9.19 | 4.09 | 22.24 | 3.66 | 26.25 | 12.45 | 36.08 | 23.75 | 13.97 | 7.61 | 31.08 | 9.48 | 0.04 |
| 26 | GlycA | 44.53 | 5.74 | 35.12 | 6.94 | 44.37 | 7.37 | 33.89 | 5.20 | 37.83 | 6.95 | 28.27 | 3.53 | 45.58 | 19.57 | 37.73 | 5.75 | 0.01 |
| 27 | glutamine | 11.70 | 2.71 | 10.09 | 1.64 | 10.59 | 1.47 | 9.68 | 1.72 | 10.25 | 1.35 | 9.05 | 1.16 | 12.29 | 2.78 | 10.38 | 2.46 | 0.18 |
| 28 | methionine | 9.39 | 2.41 | 10.14 | 1.29 | 7.23 | 1.50 | 8.90 | 1.52 | 7.40 | 1.88 | 8.56 | 2.62 | 8.61 | 1.04 | 8.27 | 2.05 | 0.11 |
| 29 | butyrate | 2.99 | 1.70 | 3.62 | 0.88 | 2.30 | 0.80 | 3.00 | 0.25 | 3.10 | 0.85 | 3.51 | 1.21 | 2.64 | 0.76 | 2.56 | 1.52 | 0.45 |
| 30 | acetoin | 3.14 | 1.48 | 2.57 | 1.01 | 4.04 | 1.06 | 4.48 | 1.32 | 5.87 | 1.98 | 4.21 | 1.26 | 4.89 | 1.16 | 3.60 | 1.69 | 0.004 |
| 31 | acetone | 2.40 | 0.99 | 1.85 | 0.73 | 3.28 | 0.55 | 2.91 | 0.74 | 3.76 | 1.68 | 2.29 | 0.73 | 3.33 | 1.18 | 2.07 | 1.06 | 0.016 |
| 32 | 2-aminoadipate | 4.68 | 2.07 | 4.37 | 1.48 | 7.39 | 2.80 | 5.59 | 0.65 | 5.35 | 1.34 | 4.06 | 0.28 | 5.37 | 1.52 | 4.05 | 2.46 | 0.05 |
| 33 | acetoacetate | 1.22 | 0.67 | 1.32 | 0.46 | 1.37 | 0.70 | 1.37 | 0.62 | 1.57 | 0.27 | 1.61 | 0.28 | 2.05 | 0.51 | 1.63 | 0.78 | 0.30 |
| 34 | glutamate | 21.69 | 5.85 | 26.24 | 2.43 | 15.30 | 3.76 | 20.92 | 5.89 | 20.59 | 6.21 | 22.53 | 7.78 | 19.30 | 6.32 | 23.18 | 6.15 | 0.06 |
| 35 | succinate | 2.03 | 3.63 | 0.66 | 0.45 | 0.25 | 0.23 | 0.93 | 0.55 | 0.73 | 0.37 | 0.87 | 0.33 | 0.50 | 0.53 | 0.57 | 0.31 | 0.56 |
| 37 | 2-oxocaproate | 0.26 | 0.35 | 0.39 | 0.35 | 0.23 | 0.55 | 0.50 | 0.26 | 0.49 | 0.38 | 0.60 | 0.59 | 0.41 | 0.43 | 0.48 | 0.49 | 0.81 |
| 40 | 2-oxovalerate | 0.44 | 0.44 | 0.34 | 0.36 | 0.35 | 0.52 | 0.27 | 0.17 | 0.46 | 0.74 | 0.48 | 0.30 | 0.39 | 0.25 | 0.25 | 0.24 | 0.98 |
| 41 | dimethylamine | 0.34 | 0.20 | 0.16 | 0.10 | 0.47 | 0.45 | 0.19 | 0.10 | 0.28 | 0.32 | 0.26 | 0.21 | 0.28 | 0.21 | 0.78 | 1.11 | 0.31 |
| 42 | sarcosine | 0.76 | 0.36 | 0.46 | 0.23 | 0.72 | 0.46 | 0.39 | 0.09 | 0.75 | 0.86 | 0.48 | 0.24 | 0.79 | 0.26 | 3.03 | 5.60 | 0.33 |
| 43 | aspartate | 5.41 | 3.82 | 4.61 | 1.99 | 2.17 | 1.24 | 2.54 | 0.64 | 3.95 | 1.60 | 3.93 | 1.38 | 2.87 | 0.91 | 3.12 | 1.91 | 0.11 |
| 45 | trimethylamine | 0.54 | 0.44 | 0.89 | 0.57 | 0.43 | 0.39 | 0.89 | 0.83 | 0.54 | 0.50 | 1.12 | 1.31 | 1.35 | 2.07 | 1.63 | 2.37 | 0.62 |
| 46 | asparagine | 1.28 | 0.89 | 1.02 | 0.80 | 1.46 | 1.11 | 1.47 | 0.82 | 1.20 | 0.90 | 2.55 | 1.69 | 2.20 | 1.10 | 1.47 | 1.62 | 0.30 |
| 47 | 2-oxoglutarate | 1.19 | 0.67 | 0.99 | 0.32 | 1.28 | 0.57 | 0.78 | 0.26 | 1.40 | 0.77 | 1.27 | 0.61 | 2.05 | 0.72 | 1.10 | 0.39 | 0.05 |
| 48 | lysine | 7.23 | 2.25 | 7.23 | 1.82 | 6.45 | 2.43 | 5.85 | 1.63 | 6.44 | 2.92 | 6.37 | 2.85 | 8.76 | 1.79 | 8.06 | 3.05 | 0.53 |
| 49 | creatine | 2.19 | 0.84 | 2.09 | 0.42 | 2.11 | 1.03 | 1.36 | 0.61 | 1.79 | 0.74 | 1.76 | 0.97 | 3.25 | 1.88 | 2.74 | 1.22 | 0.11 |
| 51 | ornithine + tyrosine | 2.80 | 2.03 | 2.75 | 1.54 | 4.89 | 6.77 | 4.74 | 5.47 | 11.04 | 2.66 | 8.75 | 2.39 | 11.33 | 5.58 | 12.48 | 4.53 | 0.0001 |
| 53 | choline | 0.42 | 0.23 | 0.56 | 0.19 | 0.35 | 0.33 | 0.21 | 0.16 | 0.33 | 0.23 | 0.38 | 0.26 | 0.53 | 0.34 | 0.36 | 0.20 | 0.31 |
| 55 | proline | 2.20 | 1.71 | 2.92 | 0.56 | 0.64 | 0.62 | 0.93 | 0.37 | 2.21 | 1.28 | 1.75 | 1.56 | 1.18 | 1.41 | 2.38 | 0.69 | 0.01 |
| 56 | methanol | 1.17 | 1.18 | 0.60 | 0.14 | 0.29 | 0.20 | 0.29 | 0.15 | 1.49 | 1.31 | 0.70 | 0.28 | 0.95 | 1.86 | 0.67 | 0.39 | 0.30 |
| 57 | taurine | 4.66 | 1.58 | 5.77 | 2.41 | 6.51 | 3.70 | 9.31 | 4.46 | 5.79 | 1.09 | 9.41 | 1.36 | 8.93 | 6.03 | 7.00 | 5.75 | 0.14 |
| 59 | ribose | 2.39 | 1.61 | 3.93 | 1.53 | 5.72 | 2.34 | 5.65 | 1.21 | 4.09 | 2.08 | 4.79 | 0.95 | 3.53 | 1.83 | 6.17 | 1.92 | 0.004 |
| 60 | glycerol | 2.45 | 1.34 | 3.45 | 2.55 | 3.85 | 2.08 | 3.09 | 0.74 | 4.42 | 1.60 | 4.11 | 1.78 | 2.66 | 1.73 | 7.57 | 4.67 | 0.017 |
| 61 | glycine | 9.92 | 2.03 | 11.52 | 2.93 | 8.68 | 4.08 | 9.05 | 2.41 | 11.78 | 3.67 | 11.70 | 1.66 | 9.93 | 4.73 | 12.08 | 7.28 | 0.64 |
| 63 | threonine | 1.87 | 1.40 | 2.98 | 1.67 | 3.26 | 1.96 | 3.87 | 1.26 | 2.88 | 0.83 | 4.30 | 1.04 | 3.41 | 1.50 | 4.77 | 2.24 | 0.05 |
| 67 | 2-aminobutyrate | 1.51 | 1.60 | 3.05 | 1.36 | 1.99 | 1.42 | 4.99 | 2.81 | 3.25 | 2.69 | 2.92 | 1.31 | 3.21 | 1.81 | 4.83 | 3.89 | 0.09 |
| 68 | glucose | 12.01 | 4.23 | 17.57 | 4.72 | 8.06 | 8.23 | 23.51 | 13.76 | 14.62 | 9.28 | 15.41 | 5.94 | 15.86 | 9.11 | 13.35 | 7.64 | 0.10 |
| 69 | N-acetylglucosamine | 0.94 | 0.74 | 1.98 | 0.91 | 0.98 | 1.09 | 2.03 | 0.52 | 1.46 | 1.38 | 1.54 | 1.05 | 1.97 | 1.44 | 1.45 | 1.51 | 0.40 |
| 70 | creatine | 2.16 | 1.36 | 2.92 | 1.02 | 1.63 | 1.26 | 2.19 | 0.80 | 2.66 | 1.73 | 2.00 | 1.24 | 3.43 | 2.34 | 3.28 | 1.58 | 0.37 |
| 72 | creatine-P | 1.99 | 1.50 | 2.66 | 1.00 | 0.93 | 1.04 | 2.08 | 0.58 | 2.19 | 1.92 | 2.36 | 1.13 | 1.94 | 0.88 | 2.59 | 0.98 | 0.34 |
| 73 | hippurate | 3.59 | 2.73 | 5.80 | 2.03 | 2.11 | 2.14 | 4.14 | 1.32 | 4.61 | 3.92 | 4.57 | 2.40 | 3.68 | 1.59 | 5.00 | 3.24 | 0.31 |
| 82 | adenosine | 0.22 | 0.45 | 0.38 | 0.61 | 0.14 | 0.12 | 0.18 | 0.16 | 0.24 | 0.19 | 0.22 | 0.16 | 0.27 | 0.31 | 0.33 | 0.38 | 0.95 |
| 83 | inosine | 0.25 | 0.64 | 0.18 | 0.39 | 0.12 | 0.18 | 0.19 | 0.21 | 0.26 | 0.19 | 0.05 | 0.03 | 0.24 | 0.31 | 0.22 | 0.17 | 0.98 |
| 84 | thymidine | 0.55 | 0.42 | 0.24 | 0.19 | 0.23 | 0.22 | 0.34 | 0.17 | 0.79 | 0.97 | 0.29 | 0.25 | 0.83 | 0.60 | 0.28 | 0.07 | 0.13 |
| 85 | urocanate | 0.78 | 0.90 | 0.57 | 0.41 | 0.46 | 0.40 | 0.51 | 0.44 | 1.15 | 0.93 | 0.43 | 0.30 | 0.92 | 1.62 | 0.93 | 0.50 | 0.72 |
| 86 | fumarate | 0.14 | 0.15 | 0.04 | 0.04 | 0.07 | 0.07 | 0.03 | 0.04 | 0.13 | 0.14 | 0.02 | 0.03 | 0.21 | 0.38 | 0.04 | 0.04 | 0.35 |
| 87 | N-acetyltyrosine | 0.67 | 0.50 | 0.69 | 0.59 | 0.53 | 0.50 | 0.53 | 0.27 | 1.07 | 0.70 | 1.00 | 0.59 | 1.30 | 0.42 | 0.82 | 0.78 | 0.27 |
| 88 | tyrosine | 4.71 | 1.46 | 5.40 | 1.16 | 3.08 | 1.44 | 4.45 | 1.15 | 4.05 | 1.23 | 4.30 | 1.91 | 4.66 | 1.01 | 4.86 | 2.68 | 0.29 |
| 91 | phenylalanine | 18.00 | 5.54 | 17.85 | 3.68 | 12.96 | 5.78 | 14.37 | 4.09 | 16.18 | 7.09 | 13.46 | 5.92 | 18.75 | 6.80 | 16.62 | 8.35 | 0.57 |
| 92 | uracil | 0.87 | 0.46 | 1.53 | 0.49 | 0.94 | 0.93 | 1.10 | 0.60 | 1.18 | 0.80 | 1.32 | 0.61 | 0.91 | 0.54 | 1.08 | 0.56 | 0.50 |
| 93 | pyridoxine | 0.24 | 0.36 | 0.02 | 0.02 | 0.14 | 0.27 | 0.06 | 0.07 | 0.37 | 0.44 | 0.04 | 0.02 | 0.32 | 0.43 | 0.10 | 0.08 | 0.23 |
| 94 | tryptophan | 0.77 | 0.41 | 0.59 | 0.27 | 0.67 | 0.54 | 0.54 | 0.19 | 1.21 | 0.89 | 0.56 | 0.25 | 0.81 | 0.75 | 1.17 | 0.55 | 0.22 |
| 95 | uridine | 0.68 | 0.49 | 0.82 | 0.58 | 0.64 | 0.22 | 0.69 | 0.32 | 0.95 | 0.63 | 0.48 | 0.21 | 0.52 | 0.44 | 0.95 | 0.66 | 0.65 |
| 96 | hypoxanthine | 1.47 | 0.93 | 2.29 | 0.86 | 1.42 | 0.66 | 1.39 | 0.54 | 2.00 | 1.24 | 1.52 | 0.71 | 0.93 | 0.83 | 1.54 | 0.80 | 0.21 |
| 97 | nicotinate | 0.36 | 0.29 | 0.31 | 0.30 | 0.52 | 0.52 | 0.20 | 0.07 | 0.29 | 0.20 | 0.24 | 0.08 | 0.26 | 0.34 | 0.33 | 0.20 | 0.72 |
| 98 | 2-deoxyadenosine | 0.26 | 0.27 | 0.07 | 0.11 | 0.19 | 0.23 | 0.06 | 0.04 | 0.18 | 0.13 | 0.03 | 0.03 | 0.21 | 0.26 | 0.14 | 0.14 | 0.26 |
| 99 | formate | 0.52 | 0.48 | 0.57 | 0.56 | 0.38 | 1.97 | 1.97 | 0.84 | 1.08 | 0.88 | 1.05 | 1.00 | 1.68 | 1.05 | 1.00 | 1.68 | 0.30 |

**Supplementary Table S3.** Relative abundances (%) at OTU level between ND and HFD colon.

**Supplementary Table S4.** Relative abundances (%) at OTU level between ND and HFD caecum.

**Supplementary Table S5.** Relative abundances (%) at OTU level between ND and 3xtg colon.

**Supplementary Table S6.** Relative abundances (%) at OTU level between ND and 3xtg caecum.

**
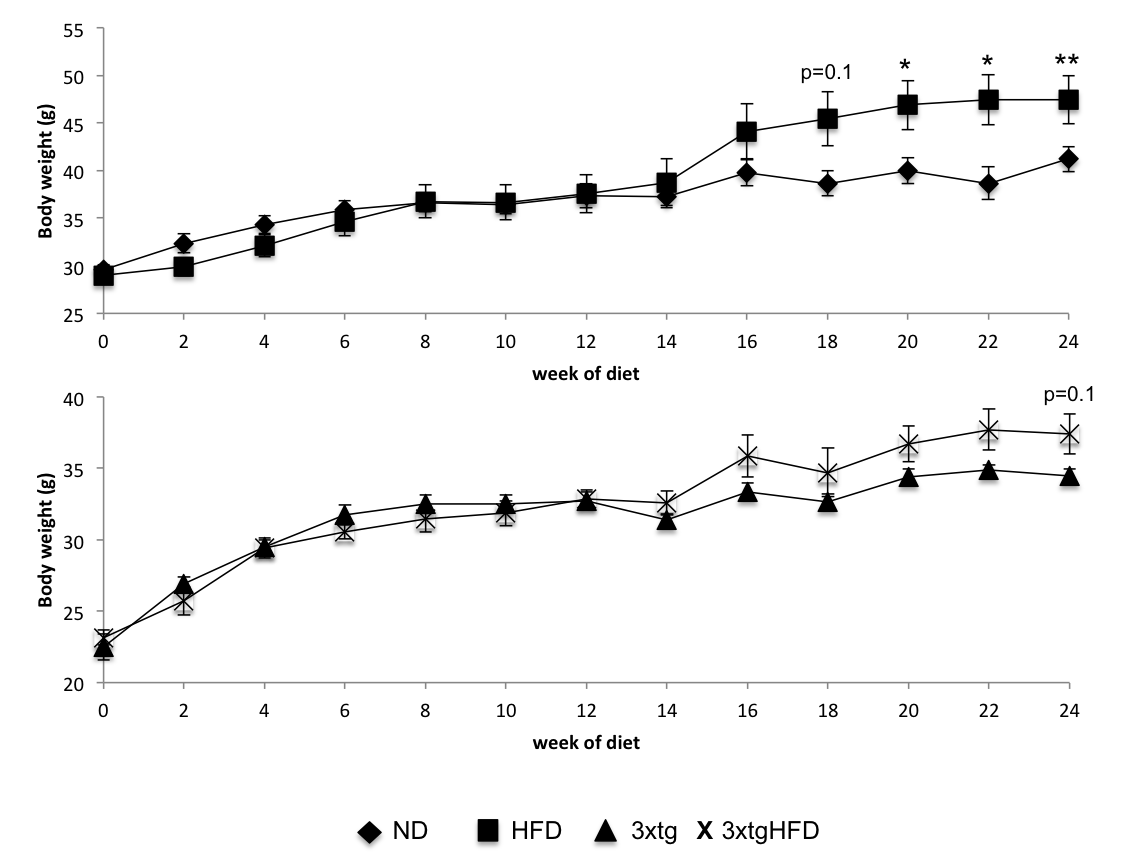
**

**Supplementary Figure S1.** Body weight curves up to 8 months of age. Mann-Withney U statistical test. Data are mean ± standard error of the mean *p<0.05, **p<0.01.


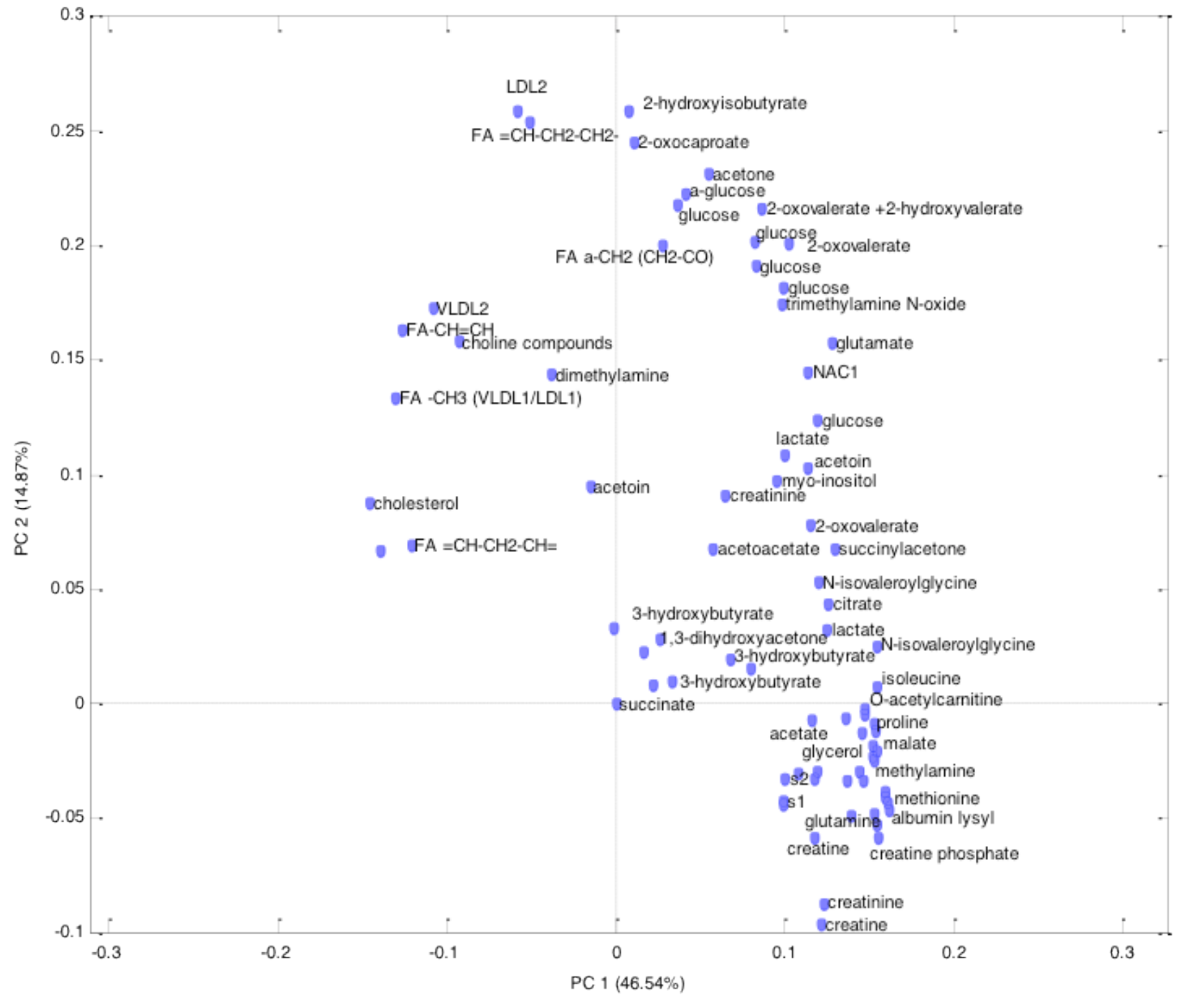


**Supplementary Figure S2.** Distribution of metabolites that explain group differences described in Fig.1-3.


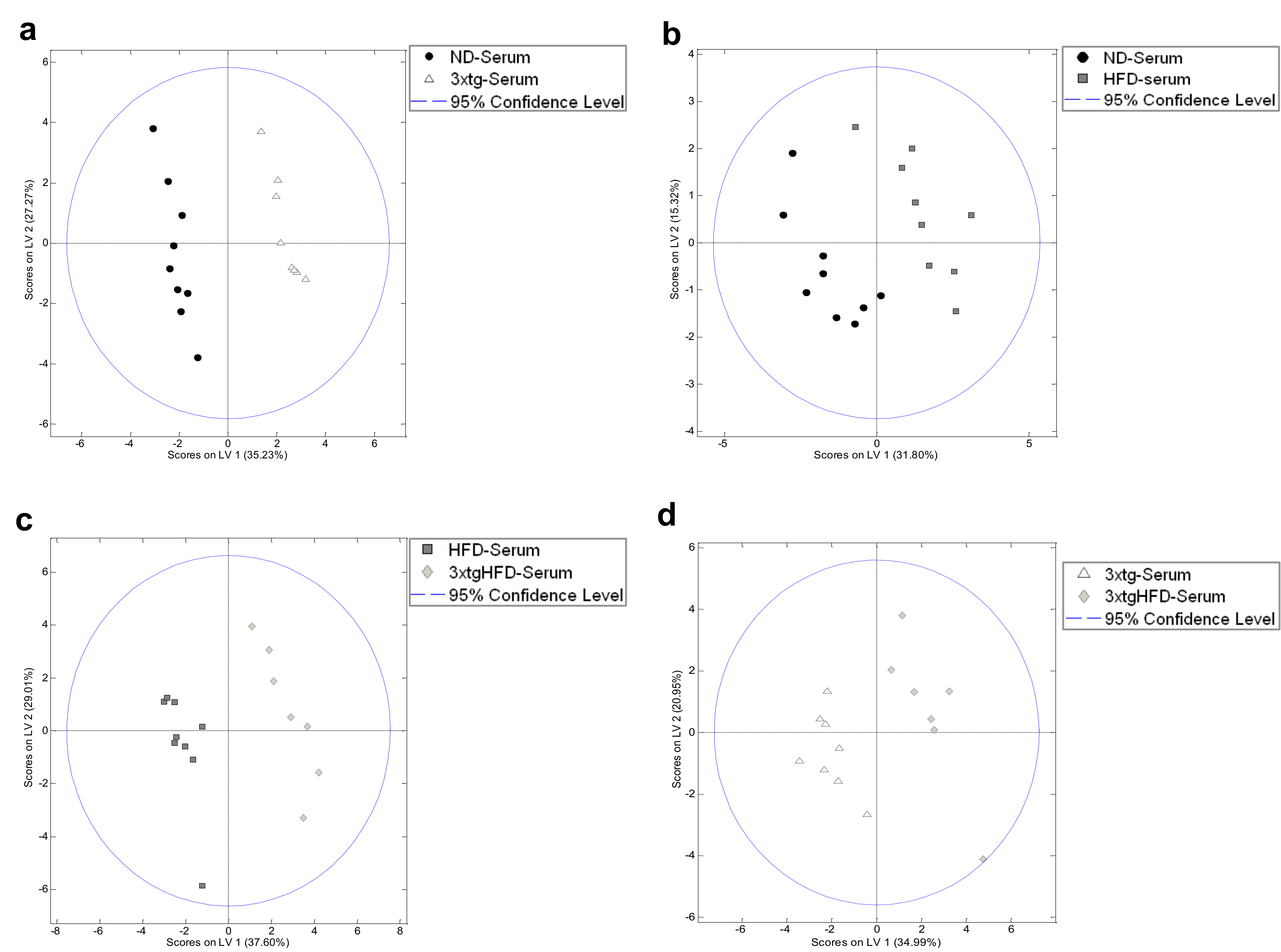


**Supplementary Figure S3.** PLS-DA scores plot based on the serum metabolomic profile to discriminate control vs 3xtg genotype under normal diet (a); normal diet vs high-fat diet on control animals (b); control vs 3xtg genotype on high-fat diet (c); normal diet vs high-fat diet on 3xtg animals (d).


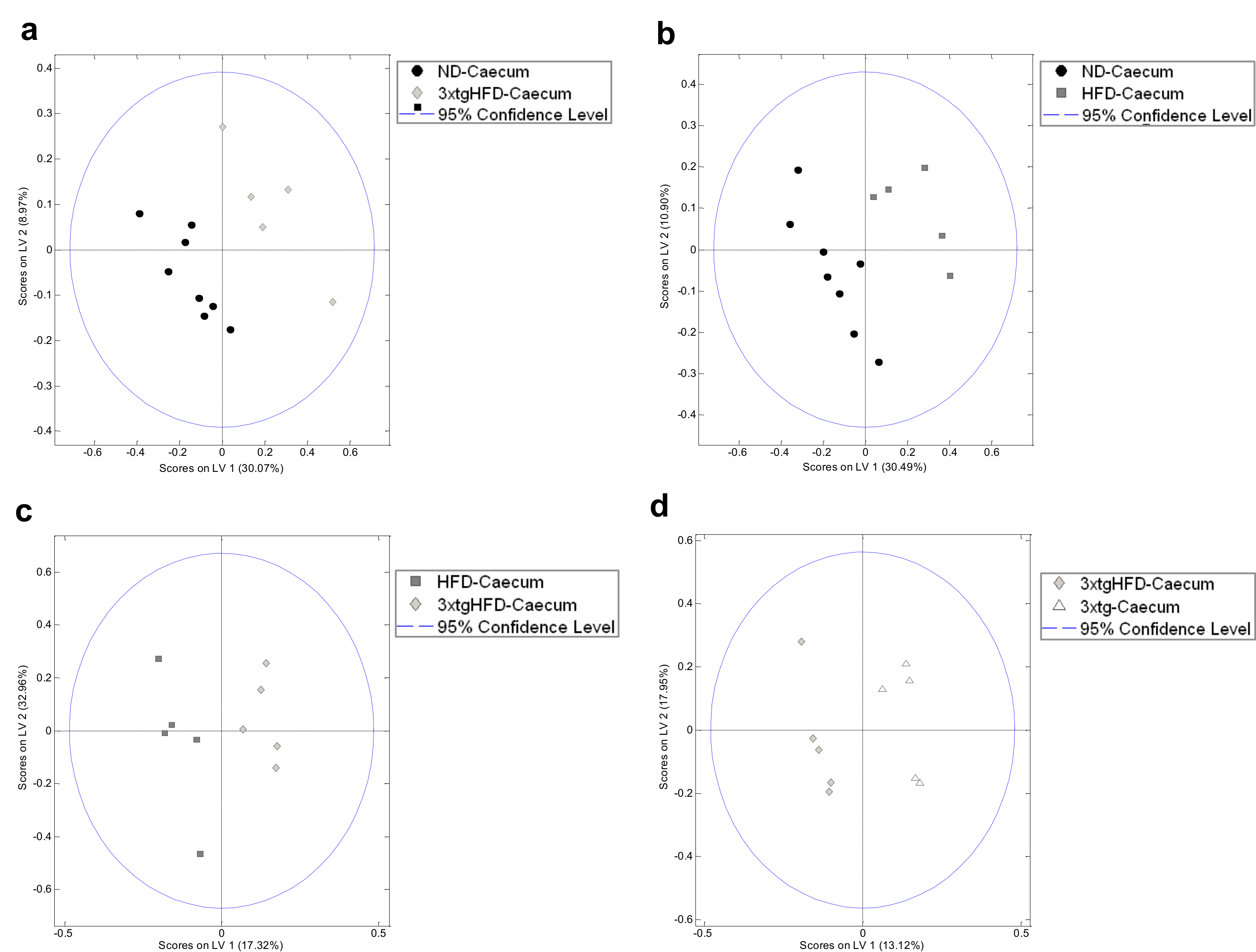


**Supplementary Figure S4.** PLS-DA scores plot based on the caecum fecal extract metabolomic profile to discriminate control vs 3xtg genotype under normal diet (a); normal diet vs high-fat diet on control animals (b); control vs 3xtg genotype on high-fat diet (c); normal diet vs high-fat diet on 3xtg animals (d).


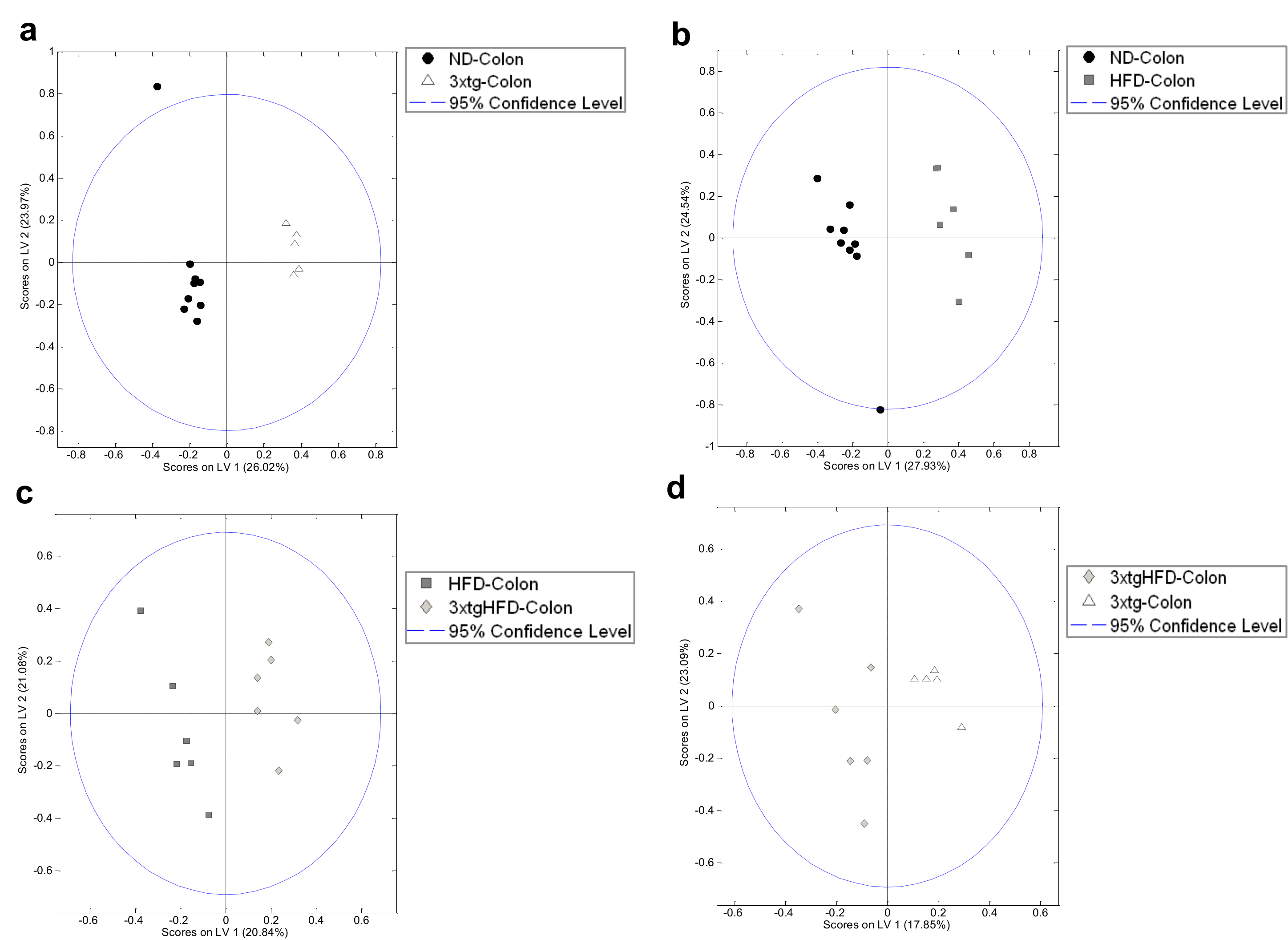


**Supplementary Figure S5.** PLS-DA scores plot based on the colon fecal extract metabolomic profile to discriminate control vs 3xtg genotype under normal diet (a); normal diet vs high-fat diet on control animals (b); control vs 3xtg genotype on high-fat diet (c); normal diet vs high-fat diet on 3xtg animals (d).

**
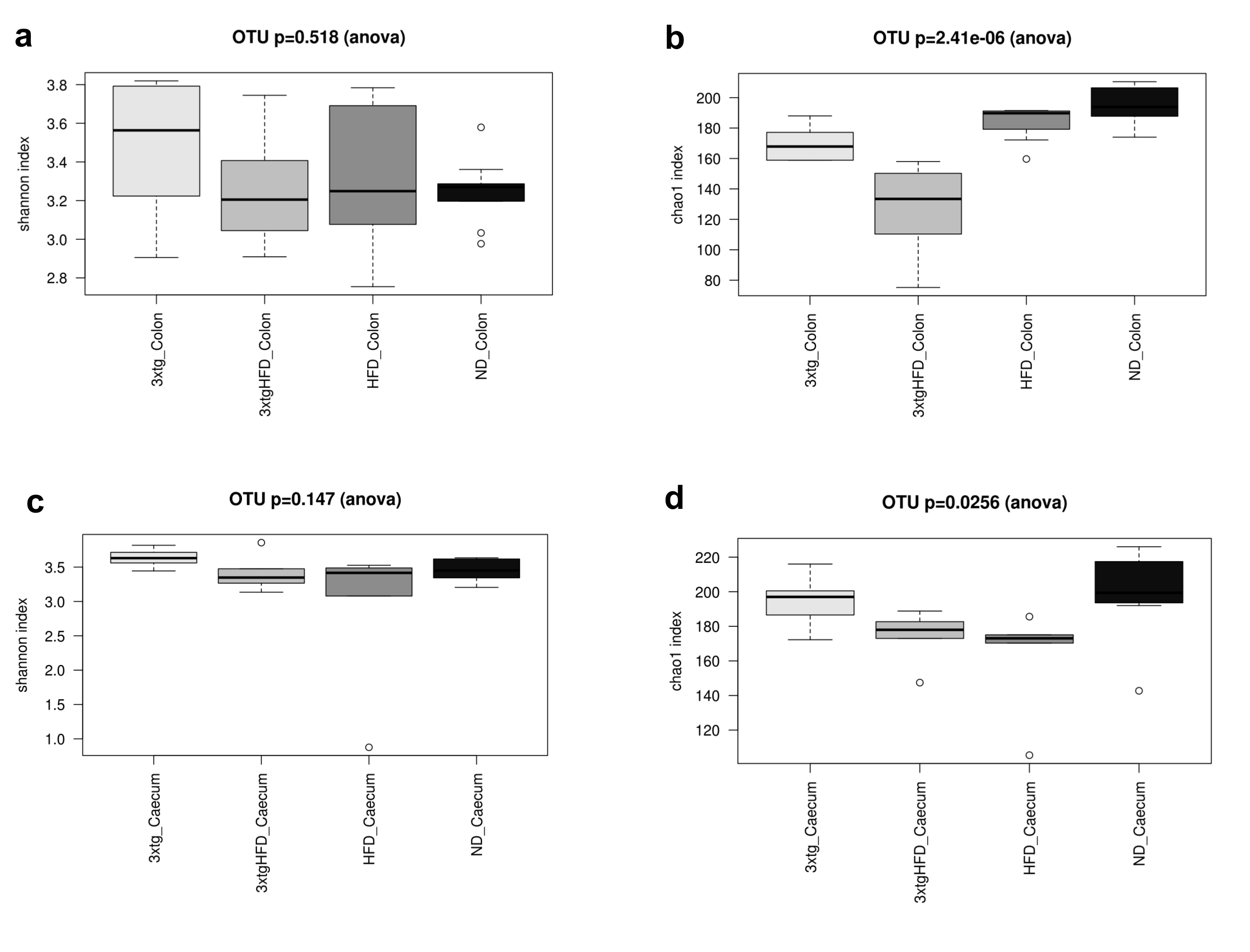
**

**Supplementary Figure S6.** Alpha-diversity plots in colon (a. Shannon index; b. Chao1 index) and caecum (c. Shannon index; d. Chao1 index).


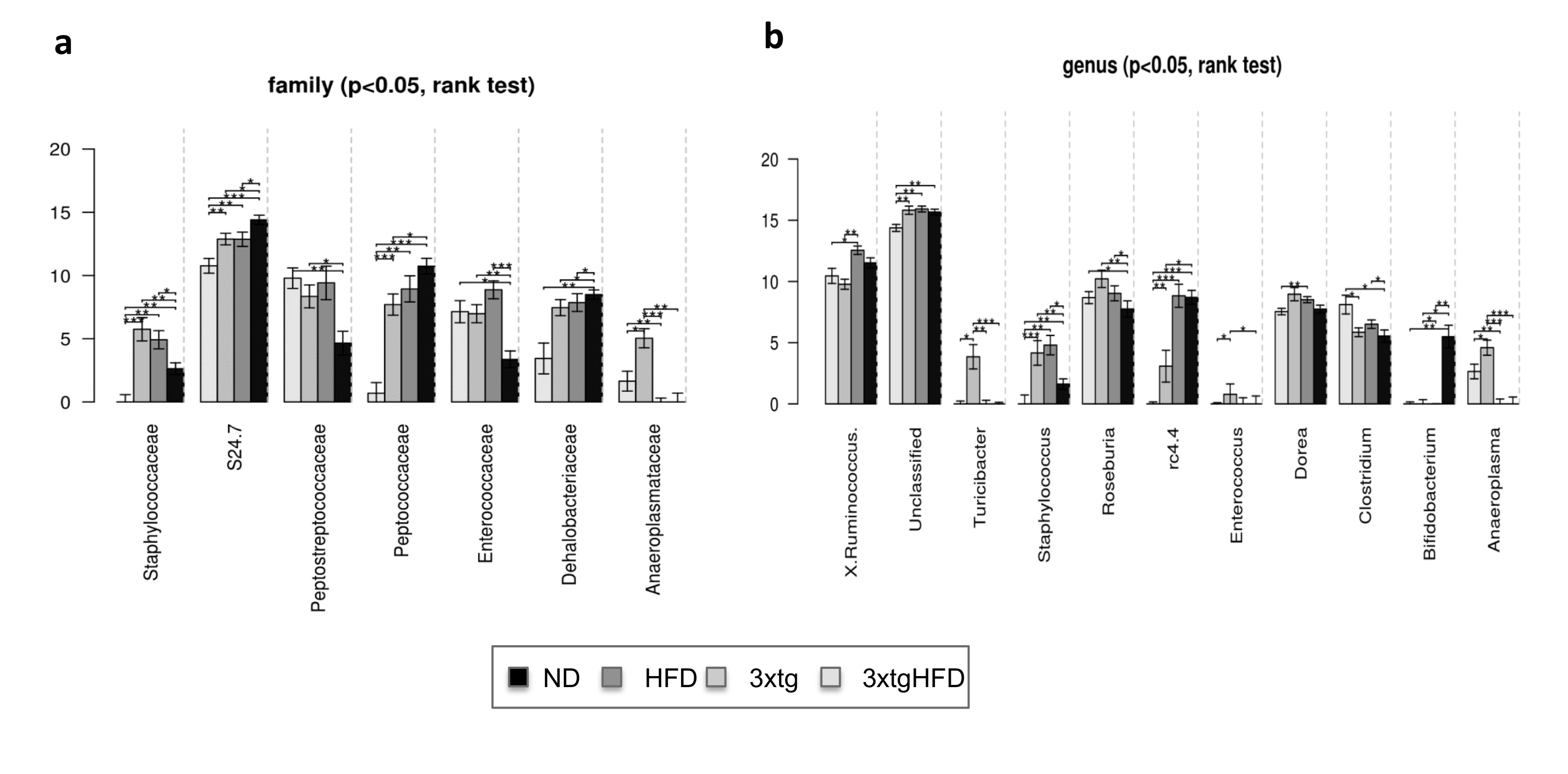


**Supplementary Figure S7.** **HFD consumption and 3xtg background significantly modify the bacterial abundances in the gut microbiome.** Relative abundance (%) of the top families (a) and genera (b) in the whole studied population. Significantly different taxa between groups are shown as bar chart (p<0.05. Rank Test). Standard error is depicted by error bars. Pair-wise comparisons are done by t-test. *p<0.05, **p<0.01.
